# Multilayered Prioritization of Graph Attention Autoencoder–Derived Drug Repurposing Candidates for COVID-19

**DOI:** 10.3390/ph19101522

**Published:** 2026-09-25

**Authors:** Yusuke Nakayama, Shingo Tsuji, Koji Yamamoto, Juran Kato-Suzuki, Kouichi Hosomi

**Affiliations:** 1 GEXVal Inc., Shonan iPark 2-26-1 Muraoka Higashi, Fujisawa-shi 251-8555, Japan; juran.kato@gexval.com; 2 Research Center for Advanced Science and Technology (RCAST), The University of Tokyo, 4-6-1 Komaba, Meguro-ku, Tokyo 153-8904, Japan; tsuji@genome.rcast.u-tokyo.ac.jp; 3 Institute for AI and Beyond, The University of Tokyo, 2-11-16 Yayoi, Bunkyo-ku, Tokyo 113-0032, Japan; 4 Independent Researcher, Tsukuba-shi 305-0817, Japan; koji.yamamoto@metcoding.com; 5 Faculty of Pharmacy, Kindai University, 3-4-1, Kowakae, Higashi-Osaka 577-8502, Japan; hosomi@phar.kindai.ac.jp

**Keywords:** drug repurposing, drug repositioning, COVID-19, SARS-CoV-2, graph attention autoencoder, knowledge graph, disproportionality analysis, real-world evidence, gene expression, hypothesis generation

## Abstract

**Background/Objectives:** The COVID-19 pandemic highlighted the critical need for drug repurposing. While computational graph-based approaches show promise, single analytical methods often fail to capture the complex pathological mechanisms needed for clinical translation. We aimed to prioritize COVID-19 repurposing candidates by combining computational prediction with orthogonal lines of evidence. **Methods:** We developed a Graph Attention Autoencoder (GATE) framework with multilayered evaluation integrating computational prediction, real-world data, and phenome analyses. Network-based embeddings identified candidates through latent-space similarity within biomedical knowledge graphs. Clinical relevance was assessed by disproportionality analysis (DPA) of an adverse event reporting system, and biological plausibility through gene expression and pathway profiling. **Results:** Our GATE framework identified 16 candidate drug entities, including 12 already used or recognized in the context of COVID-19 or its symptoms, consistent with the model recovering known drug–disease associations. The remaining four had minimal prior COVID-19 associations (cilastatin, megestrol, drotrecogin alfa, and ethacrynic acid). Of these four, the multilayered evaluation prioritized two: DPA showed significant inverse reporting associations for COVID-19-related adverse event terms for cilastatin (reporting odds ratio [ROR]: 0.19, 95% confidence interval [CI]: 0.09–0.43) and megestrol (ROR: 0.63, 95% CI: 0.42–0.94). Pathway profiling indicated that cilastatin shares molecular signatures with drugs investigated in COVID-19 clinical trials and megestrol with compounds reported as COVID-19 therapeutic candidates; gene expression analyses suggest cilastatin may exert antiviral and anti-inflammatory effects through DPEP1 inhibition. **Conclusions:** By integrating GATE-based prediction, real-world evidence, and phenome-level pathway profiling, this study enabled multidimensional candidate prioritization that single analytical methods do not provide. These findings, including the proposed mechanisms of action, are computational predictions that await experimental confirmation. They nonetheless provide mechanistic working hypotheses that may streamline subsequent preclinical studies. Prioritizing clinically approved drugs with established safety profiles may facilitate subsequent clinical evaluation, although the risks specific to each drug would need to be weighed. The framework offers a generalizable strategy for rapid candidate identification during pandemics and diseases with unmet therapeutic needs.

## 1. Introduction

The spread of infectious diseases and the emergence of new pathologies have particularly increased the demand for treatments targeting diseases and conditions with limited therapeutic options. The COVID-19 pandemic highlighted critical gaps in rapid therapeutic development, demonstrating the urgent need for drug repurposing strategies [1]. While traditional drug development requires over 10 years and billions of dollars [2,3,4], with approximately 10% success rates in clinical development [5], drug repurposing offers accelerated timelines and higher success probabilities due to established safety and pharmacological profiles [6,7]. Indeed, several computational approaches have successfully identified COVID-19 therapeutics, including baricitinib through network-based repositioning [8], validating the potential of systematic computational drug discovery.

Computational drug repurposing leverages public databases and bioinformatics to propose novel therapeutic candidates [6]. Graph structure-based approaches—which model relationships between diseases, drugs, and genes as interconnected networks—have emerged as powerful tools for capturing biological complexity. Ritonavir (a component of Paxlovid) and atorvastatin were successfully identified as potential COVID-19 treatments through network-derived relationship analysis, highlighting how graph-based representations can serve as a powerful foundation for systematically uncovering repurposing opportunities [9].

In graph structure-based drug repurposing, it is common to convert graph nodes and edges into vector representations through embedding. Graph embedding methods have been applied to recommendation systems in web stores and other applications [10]. Building upon these foundations, Graph Neural Networks (GNNs), which employ neural networks for graph embedding, have been proposed [11] and have become widely used.

While various GNN methods exist, Graph Attention Networks (GATs) that utilize attention mechanisms have been widely adopted [12]. The attention mechanism is a technology used in Transformer Architecture, which forms the foundation of generative AI using large language models such as ChatGPT, and supports today’s revolutionary AI evolution [13]. Recently, these GATs have increasingly been applied to drug repurposing research [14]. However, existing GNN approaches face critical limitations when confronting complex pathological mechanisms; moreover, predictions from any single computational method may be insufficient on their own and require complementary, orthogonal lines of evidence, and ultimately experimental validation, before they can guide therapeutic development.

In fact, COVID-19 pathogenesis involves complex, multifaceted mechanisms encompassing viral replication, host immune responses, and cellular metabolic processes. Approximately 200 existing drugs may target SARS-CoV-2-associated pathways, spanning diverse targets including viral proteins (spike protein, papain-like protease (PLpro), 3-chymotrypsin-like protease (3CLpro), RNA-dependent RNA polymerase (RdRp), and helicase [15]) and host cellular machinery (mTOR-PI3K pathway [16], autophagy [17], vesicular transport systems [18], and immune response [19]). This molecular complexity presents a fundamental challenge; single analytical approaches may be insufficient for adequate exploration of therapeutic candidates.

To address this challenge, we developed a comprehensive multilayered analytical framework that integrates a Graph Attention Autoencoder (GATE) with evaluation using real-world evidence and phenome data. Rather than relying on a single line of evidence, the framework triangulates each candidate across independent, heterogeneous data sources. This integrated strategy prioritized candidate drugs that had been proposed but not investigated in depth as COVID-19 therapeutics, such as cilastatin and megestrol, and generated actionable mechanistic hypotheses for therapeutic development. The primary contribution of this work lies not in the identification of any single drug, but in the corroboration of graph-derived candidates through independent lines of evidence.

## 2. Results

### 2.1. GATE-Based Identification of Candidate Drugs for COVID-19

To systematically identify and prioritize novel drug repurposing candidates, we developed a comprehensive multilayered analytical framework that complements single-method computational prediction with orthogonal lines of evidence. Our methodology consists of three integrated phases: (1) computational identification of candidate drugs through a graph attention autoencoder (GATE) utilizing attention mechanisms and data obtained from Open Targets [20]; (2) assessment of potential clinical relevance using real-world evidence; and (3) molecular biological assessment for mechanism of action (MOA) hypothesis generation using phenome data (Figure 1). This multilayered evaluation strategy integrates complementary lines of evidence, providing more actionable insights for therapeutic development than network-based prediction alone. To demonstrate the utility of this framework, we applied it to identify and prioritize novel drug repurposing candidates for COVID-19.

Based on the latent space representation of GATE, we identified drug nodes showing high similarity to the COVID-19 node (Table 1). In total, 21 drug nodes fell within the top 500 of all ranked nodes. In interpreting the results, we considered different salt or derivative forms of the same compound (e.g., cilastatin and cilastatin sodium) as representing a single drug entity, which reduced these 21 nodes to 16 distinct drug entities. Among the 16 drug entities identified, 12 are already used in the clinical management of COVID-19 or its symptoms, or are otherwise recognized as being used in that context, consistent with the model recovering established, low-novelty drug–disease relationships. The remaining four drugs (cilastatin, megestrol, drotrecogin alfa, and ethacrynic acid) had minimal prior association with COVID-19, representing candidates that are less documented and have received less attention as COVID-19 therapeutics. Notably, these candidate drugs and COVID-19 were not directly connected in the original knowledge graph (Figure 2 and Figures S1–S3); their proximity in the latent space reflects similarity in node features and in the information their neighborhoods contain rather than the presence of a direct edge.

To further evaluate the findings, we investigated the association scores between COVID-19 and the target genes of each candidate drug using the Open Targets Platform, an integrated drug discovery database. Association scores are comprehensive relatedness indicators based on information in the literature, genetic evidence, experimental data, and other sources. This analysis revealed that all four candidate drugs showed low association scores with COVID-19 (Table 1), indicating that the association between these drugs and COVID-19 is weakly represented in Open Targets. A low association score in a single resource is not by itself evidence of novelty, and three of the four candidates (cilastatin, megestrol, and ethacrynic acid) have been proposed as COVID-19 candidates in earlier computational studies. Our top candidate, cilastatin, had an association score of 0.01, which is markedly lower than scores (0.068–0.609) of drugs that are known to have effects on COVID-19.

### 2.2. Retrieval Performance and Information Leakage

The candidates above were selected by proximity in the GATE latent space; to establish what that ranking recovers, we evaluated it on repoDB [21], using the 807 approved indications across 239 diseases that carry no corresponding edge in the graph (hereafter unlinked indications). Applying the same procedure and cutoff, GATE recovered 44.0% of them within the top 500 of all 27,644 nodes; the input text embedding from which the latent space is derived recovered 21.4% and common neighbors 17.8%, while preferential attachment, which ranks by degree alone, recovered none because drug nodes carry lower degree than disease and gene nodes (Figure 3A, Table S1). On the ranking over all nodes, GATE recovered more of the unlinked indications than a node2vec embedding trained on the same graph up to a rank of about 330, and fewer beyond it; at the top-500 cutoff, the two recover 44.0% and 49.6% (Figure 3A, Table S1). On the ranking over the 4314 drug nodes alone, the two do not cross, and GATE recovers more at every cutoff below the full list, 80.3% against 68.2% at the top 500, whereas preferential attachment is competitive at the very top (Recall@10 0.190 against 0.206) though it falls behind at greater depth (Recall@100 0.333 against 0.627) (Figure 3B, Table S1). The median unlinked indication falls within the top 1.41% of the 4314 drug nodes for GATE and 4.24% for node2vec, whereas over all 27,644 nodes the corresponding values are 3.66% and 1.87% (Table S1). Discrimination of individual drug–disease pairs was weaker than that of the input text embedding on every negative set, most markedly against the clinically hard negatives (area under the receiver operating characteristic curve (AUROC) 0.573 against 0.701), and pairwise AUROC differed by 0.056 between indications present as training edges and those absent, indicating that the ranking reflects both the existing edges and the biological knowledge of nodes (Table S2).

For the COVID-19 query itself, no unlinked indications exist. Of the 17 drugs used or evaluated in COVID-19 that are present in the graph, the median rank was 135 among the 4314 drug nodes, and eight fell within the top 100, but only inhaled budesonide (361st) fell within the top 500 of all nodes used here (Table S3). Recovery and enrichment varied smoothly across cutoffs, with no discontinuity near 500; at the top-500 cutoff, the recovery of 44.0% corresponds to a 24.3-fold enrichment over the 1.81% expected by chance (Table S4). For instance, cilastatin and megestrol are selected at any cutoff from 400 to 2000, whereas drotrecogin alfa (412th) and ethacrynic acid (492nd) lie close to the cutoff and would not be selected by a stricter one (Table S5A). We also examined whether COVID-19-specific information could have reached the representation. No edge connects the COVID-19 node to any drug node, and the node reaches the graph only through 462 gene edges, to which the single attention layer confines the structural contribution. Ten of the twenty-one retrieved drug nodes share at least one gene neighbor with the COVID-19 node, while the four candidates carried forward share none and lie at graph distance three (Table S5A), and of the 20 node descriptions among 27,633 that mention COVID-19 or SARS-CoV-2, none belongs to a retrieved drug (Table S6).

Training was repeated from two further independent initializations, and the same candidate-identification procedure was applied to each; 14 and 19 of the 21 drug nodes reported here recurred in the two repeat runs, respectively, and cilastatin and megestrol were retained in all three models. Recovery of the unlinked indications followed the same shape in all three models but differed in level, reaching 44.0% for the model reported here and 58.5% and 48.6% for the two repeat runs within the top 500 of all nodes (Figure S4); the model reported here was the first of the three to be trained and was not selected on performance.

### 2.3. Disproportionality Analysis of Candidate Drugs in FAERS

We further analyzed the four drugs identified by the GATE approach using real-world adverse event (AE) data from the FDA Adverse Event Reporting System (FAERS) to examine potential associations with COVID-19-related AE terms by disproportionality analysis (DPA). In this DPA, the reporting odds ratio (ROR) and its 95% confidence interval (CI) quantify whether COVID-19-related AE terms are reported disproportionately more or less often for a given drug relative to all other drugs in the database; an ROR < 1 indicates an inverse reporting association, and a 95% CI upper limit < 1 indicates that this association is statistically significant at the reporting level. Both cilastatin and megestrol showed significant inverse reporting associations for COVID-19-related AE terms, with RORs of 0.19 (95% CI: 0.09–0.43) and 0.63 (95% CI: 0.42–0.94), respectively (Figure 4).

To examine whether the observed inverse associations were specific to each drug, we conducted DPA for drugs in the same class as each candidate drug or for drugs that are often co-administered with the candidate drug. Cilastatin is available only as a fixed-dose combination with imipenem, a carbapenem antibiotic, and the two components cannot be separated in these data. In DPA of the carbapenem class, imipenem itself showed an inverse association comparable to that of cilastatin (ROR 0.32, 95% CI 0.19–0.53, against 0.19, 0.09–0.43), whereas meropenem was associated in the opposite direction (ROR 1.59, 95% CI 1.39–1.81) (Figure S5A,B). Among β-lactamase inhibitors, which are given with β-lactam antibiotics in comparable clinical settings, none showed a statistically significant inverse association (Figure S5C,D). Megestrol is a progesterone receptor agonist. Progesterone preparations classified as progesterone receptor agonists showed RORs around 0.5 with 95% CI upper limit less than 1, indicating inverse association trends similar to megestrol (Figure S6).

Note that drotrecogin alfa and ethacrynic acid could not be evaluated using this DPA because no reports for these drugs were present in the dataset analyzed.

### 2.4. Pathway-Profile Concordance with Known COVID-19 Candidates (LINCS 2020)

To further explore these findings at the phenome level, we conducted correlation analysis using pathway profiling with the Library of Integrated Network-Based Cellular Signatures (LINCS) 2020 for cilastatin and megestrol, the two drugs that showed statistically significant inverse associations in DPA. LINCS 2020 extends the Connectivity Map (CMAP), an established platform for comparing phenomic signatures of any given drug with those associated with specific diseases or disease therapeutics [22]. For each drug, we explored compounds with correlated pathway profiles from 17,170 compounds (241,597 profiles) using Fisher’s exact test.

The compounds showing the strongest correlations with cilastatin (Table 2) included artesunate and sirolimus, which are drugs that have undergone clinical trials for COVID-19. The analysis also revealed ipidacrine [23], ursolic acid [24,25,26], wortmannin [27,28], and thapsigargin [29,30,31], which have been reported in the literature as COVID-19 therapeutic candidates.

Similarly, the compounds showing the strongest correlations with megestrol (Table 3) included fedratinib (TG-101348) [32], AZD-6482 [23] and vorinostat [33,34,35], which have been reported in the literature as COVID-19 therapeutic candidates. None of these compounds has been evaluated in a COVID-19 clinical trial.

### 2.5. Pathway Alterations Following Cilastatin Treatment

In the LINCS 2020 data, cilastatin treatment significantly decreased the expression of 42 pathways in the HT29 cell line (Table S9). Enrichment Map analysis revealed that gene sets related to splicing, ribosome, mitochondria, DNA metabolism, and ubiquitin pathways were down-regulated (Figure 5).

## 3. Discussion

### 3.1. Screening Role of the GATE Approach and Assessment of Information Leakage

In the retrieval evaluation, GATE recovered unlinked indications for their own disease far more frequently than expected by chance, and outperformed both the input text embedding from which its latent space is derived and common neighbors, a simple measure of shared graph structure. It did not, however, separate individual drug–disease pairs, a task on which the input text embedding performed better. This suggests that the GATE latent space is suited to screening candidates for a disease rather than to predicting individual drug–disease links. Over all 27,644 nodes, GATE and node2vec recovered the unlinked indications at a similar level, well above the input text embedding and the two graph-based baselines. Because candidates are inspected from the top of a ranking downward, what matters for screening is the behavior near the top rather than the proportion eventually recovered, and the output of GATE is ahead of both among the drug nodes and within the first few hundred of all nodes. Among the drug nodes, preferential attachment, which ranks drugs by degree alone and does not use information about the queried disease, performed similarly to GATE at the very top of the ranking but fell behind further down the ranking. This suggests that degree alone may explain part of the early recovery, but not the broader recovery achieved by GATE. Ranking the drug nodes alone and ranking them among all nodes use the same cosine-similarity scores, but differ in the nodes included in the ranked list: the all-node ranking also includes disease and gene nodes, whereas the drug-only ranking excludes drugs already linked to the query disease. The difference between the two therefore reflects the composition of the ranked list rather than a change in how the drugs themselves are scored. What the non-drug nodes represent has not been examined here, and characterizing them is a direction for future work. A ranking of this kind will contain candidates for which the later phases find no support, and the multilayered framework was designed on that basis. Broad retrieval before assessment against orthogonal lines of evidence is the organizing principle of our prioritization approach.

COVID-19-specific therapeutic knowledge could have entered the GATE latent space only through the gene neighborhood of the COVID-19 node: no edge connects COVID-19 to any drug, the single attention layer confines the structural contribution to that neighborhood, and none of the descriptions of the drugs retrieved within the cutoff mention COVID-19. That neighborhood is not neutral: all 17 drugs used or evaluated in COVID-19 that are present in the graph share at least one gene with the COVID-19 node, and for most of them the shared gene is a drug target (Table S5B). Their higher-than-chance ranking for this query could therefore be read as evidence of such knowledge. The corticosteroids among them, however, ranked together, and dexamethasone, the drug with the clearest mortality benefit in COVID-19, was not placed above the others in its class. These drugs also ranked higher for 21 other diseases than for COVID-19, all of them inflammatory or autoimmune, and six of those diseases, like COVID-19, carry no edge to any of these drugs in the graph (Table S10). This suggests that their ranking may reflect a pharmacological class effect rather than knowledge of clinical outcomes, and that such knowledge contributed little to it.

### 3.2. Recovery of Latent Drug–Disease Associations by the GATE Approach

To assess whether the four candidates identified by the GATE approach had been overlooked in prior repurposing efforts, we examined whether they had been reported as COVID-19 repurposing candidates—and, where reported, investigated in depth—in existing studies. Cilastatin was reported as one of 78 candidates for SARS-CoV-2 3CLpro inhibition through docking simulation approaches, but detailed validation was not performed [36], and it has separately been proposed as a treatment strategy against COVID-19 on mechanistic grounds [37]. Megestrol was proposed as one of 34 COVID-19 therapeutic candidates through network-based approaches, though detailed investigation was not conducted [38]. Drotrecogin alfa had no existing reports; to our knowledge, this is the first report of this drug as a COVID-19 candidate. Drotrecogin alfa was, however, withdrawn from the market worldwide in 2011, after the PROWESS-SHOCK trial found no reduction in 28-day mortality in septic shock (26.4% with drotrecogin alfa against 24.2% with placebo) [39]. Ethacrynic acid was reported as a promising candidate for SARS-CoV-2 main protease (Mpro) inhibition through docking simulation approaches, with experimental validation also performed [40].

Although some candidate drugs identified by the GATE approach have been previously reported by other computational methods, those studies provided only preliminary identification without comprehensive evaluation of therapeutic potential. Notably, three of the four candidates (cilastatin, drotrecogin alfa, and ethacrynic acid) were not identified by the conventional network-based approaches we surveyed. Drotrecogin alfa, which none of those studies nominated, is the clearest example. This comparison covers a small number of published studies with differing data sources and objectives, and does not establish that these associations are undetectable by network-based methods in general. It concerns which candidates were retrieved, and is separate from whether a given candidate could be developed.

These results suggest that the GATE approach is complementary to existing methods and has the potential to efficiently discover novel relationships between drug–disease pairs. Furthermore, the identification of candidates such as ethacrynic acid, for which experimental validation has been conducted, provides additional support for the plausibility of the prioritized candidates. However, these remain computational predictions that require experimental validation.

### 3.3. Potential Clinical Relevance of Candidate Drugs from FAERS Disproportionality Analysis

For cilastatin, COVID-19-related AE terms were reported approximately 5-fold less frequently among cilastatin reports than among non-cilastatin reports, and for megestrol approximately 40% less frequently. Although these disproportionality signals are hypothesis-generating and cannot by themselves establish a causal effect, the inverse association for cilastatin was among the largest observed in this analysis, suggesting that both drugs may have potential worth investigating as COVID-19 repurposing candidates.

The carbapenem comparison suggests that the inverse reporting association observed for cilastatin is not a uniform class effect, since meropenem was associated in the opposite direction. Neither can the association be regarded as specific to cilastatin, given that imipenem showed one of similar magnitude. Since cilastatin is marketed only as a fixed-dose combination with imipenem, the two components cannot be separated in spontaneous reports, an inherent limitation of disproportionality analysis for drugs available only in combination. For megestrol, the progesterone receptor agonist comparison suggests that the observed inverse reporting association reflects a pharmacological class effect mediated through progesterone receptors rather than a signal specific to megestrol. However, more detailed analysis is needed to clearly distinguish the influence of class effects.

The inverse reporting association observed for cilastatin is consistent with previously proposed mechanisms by which this drug might reduce SARS-CoV-2 viral replication and modulate the cytokine response observed in severe COVID-19 [37]. That proposal was made on mechanistic grounds alone; the present study adds real-world reporting and phenome-level evidence bearing on the same candidate. Based on these findings, cilastatin warrants further investigation as a potential therapeutic candidate for COVID-19.

Regarding megestrol, beyond its identification in a broader computational screen [38], no literature has reported direct associations with COVID-19. However, progesterone, a drug in the same class that showed a significant inverse reporting association with COVID-19-related AE terms in our DPA, has anti-inflammatory properties, suggesting potential as a COVID-19 treatment [41,42,43]. Clinical studies on COVID-19 treatment using progesterone have been conducted (NCT04365127, NCT04865029), and in the NCT04865029 trial, progesterone administration in moderate to severe COVID-19 patients resulted in significant improvement in clinical status (a score of 1.5 points, corresponding to a 3-day reduction in oxygen supplementation and 2.5-day reduction in hospitalization duration) [44]. Given the observed class effect of progesterone receptor agonists and the clinical evidence supporting progesterone, megestrol merits evaluation to determine whether it shows similar effects in confirmatory studies.

Megestrol carries risks that are relevant to this setting. Thromboembolic, metabolic, and adrenal adverse effects are documented in the approved labeling, and adverse events occur more frequently with megestrol than with placebo [45,46]. Two of these bear directly on severe COVID-19: adrenal suppression, for which glucocorticoid cover is recommended during serious intercurrent infection, and thromboembolism, which is frequent in this population even under prophylaxis [47]. Animal toxicity data additionally point to increased susceptibility to respiratory infection [45]. These risks would require careful consideration before megestrol could be evaluated in this indication.

### 3.4. Phenome-Level Plausibility from Pathway Profiling

These phenome-level correlations suggest that both cilastatin and megestrol may have potential as COVID-19 therapeutic candidates, though the supporting evidence differs: the compounds correlated with cilastatin include drugs that have undergone COVID-19 clinical trials, whereas those correlated with megestrol have been reported only in the literature. The correlation with established COVID-19 therapeutic candidates supports the plausibility of cilastatin as a candidate worth further investigation.

### 3.5. Hypothesized Mechanisms of Action of Cilastatin

To provide mechanistic insights that could guide future preclinical studies, we sought to infer possible biological mechanisms from pathway profiling data. We argued that because pathway-level information represents a functional layer closer to biological responses than individual genes, a detailed analysis of the LINCS 2020 data can facilitate hypothesis generation regarding mechanisms of action.

Based on the pathway alterations following cilastatin treatment (Figure 5), we constructed a mechanistic hypothesis for how cilastatin might intervene in the pathophysiological processes of COVID-19 induced by SARS-CoV-2 infection (Figure 6).

Cilastatin is clinically used as a renal dehydropeptidase-I (DPEP1) inhibitor in combination with imipenem to prevent renal degradation of the antibiotic by DPEP1. Beyond this established role in antibiotic protection, our analysis of LINCS 2020 data indicated that cilastatin modulates multiple cellular pathways related to splicing, ribosomal function, and mitochondrial processes in cells. Gene sets in all three groups were among the 30 of the 42 that also satisfied a false discovery rate (FDR) < 0.05 (Table S9). DPEP1, in addition to its primary function of dipeptide hydrolysis in the kidney, also functions as a neutrophil adhesion receptor on vascular endothelial cells and is known to promote neutrophil recruitment during inflammation [48]. DPEP1 may restrict host ribosomes utilized by SARS-CoV-2 by controlling the PI3K/Akt/mTOR pathway [49], which regulates cellular protein synthesis and metabolism [52]. This may suppress viral replication through a mechanism similar to that of mTOR inhibitors such as sirolimus (rapamycin) [53].

Of particular interest, it is known that the nonstructural protein 16 (NSP16) of SARS-CoV-2 binds to U1/U2 snRNA and interferes with the host splicing mechanism [54]. Suppression of splicing-related pathways may function as a cellular stress response that limits host gene expression and potentially suppresses viral replication by restricting protein synthesis. Moreover, splicing abnormalities may activate antiviral responses via RIG-I-like receptors [50].

Beyond the splicing mechanism, cilastatin also affects mitochondrial pathways. Decreased expression of mitochondria-related genes suggests changes in cellular metabolic activity and immune signaling. These changes may contribute to balanced immune regulation by limiting energy available for viral replication while maintaining type I interferon responses through mitochondrial antiviral signaling protein (MAVS) [51].

We emphasize that this mechanistic model is a hypothesis rather than an experimental finding. The individual relationships on which it draws are supported by the published studies cited above; however, those studies did not examine cilastatin in the context of SARS-CoV-2 infection, and our own evidence is limited to transcriptomic responses measured in cancer cell lines in the absence of viral infection. These transcriptomic responses are not necessarily specific to cilastatin, since exposure to unrelated compounds might produce similar pathway changes. Establishing specificity would require testing such compounds at similar concentrations in the same cell line. Neither the assembled cascade nor its proposed antiviral and anti-inflammatory outcomes have been tested experimentally in this study. Testing whether these changes depend on DPEP1, in a respiratory-relevant model under infection, will be needed to evaluate this hypothesis.

### 3.6. Study Limitations

While our multilayered computational approach successfully identified several promising therapeutic candidates and generated mechanistic hypotheses, there are several important limitations that must be addressed.

*Limitations of the GATE approach:* The predictive performance of our knowledge graph-based method heavily depends on the comprehensiveness and quality of the base knowledge graph being used. In biomedical knowledge graphs, data bias tends to occur based on research progress and attention levels, with particularly insufficient information regarding rare diseases and drugs in early development stages. This deficiency may limit the discovery of novel drug–disease associations and reduce predictability. It also limits what can be evaluated: because COVID-19 emerged only in 2019, it is absent from earlier versions of Open Targets and has no approved indications that could be withheld, so temporal validation could not be carried out, and retrieval could not be measured for this query. Importantly, the absence of an approved drug–disease pair from the graph does not necessarily indicate a genuinely unseen association, because repoDB and the knowledge graph used here may differ in disease identifiers, disease granularity, and the indications they record as approved. Thus, the evaluation set may include both pairs that are genuinely absent and pairs that are missing for representational or curation-related reasons, which should be considered when interpreting retrieval performance. Therefore, developing comprehensive knowledge graphs that systematically incorporate data on rare diseases and emerging therapeutics will be essential for improving comprehensiveness and reducing bias in future studies.

A further limitation arises from the model itself: the latent space cannot be decomposed into its contributing sources, so the reason a drug lies close to the COVID-19 node cannot be stated. The autoencoder was trained for a fixed number of steps; as the objective is unsupervised reconstruction on a single fixed graph, no validation split was used, and no checkpoint was selected; the model reported is the state reached at the end of training. As reported in the Results, the candidate list is only partly stable across independent initializations: the two drugs taken forward were retained in all three models, whereas the wider ranking was not fully reproduced. It should therefore be read as one realization rather than a fixed list. The same constraint applies to the evaluation: assessing retrieval on indications removed from the graph before training would require a retrained model, and this remains to be done.

*Limitations of real-world data analysis:* Since FAERS is a spontaneous AE reporting system, it inherently contains problems such as reporting bias, underreporting, and selective reporting. A critical limitation emerged from our analysis of drotrecogin alfa and ethacrynic acid, which revealed that the method cannot analyze drugs with insufficient data in the database. This particularly affects drugs with few reports in the database, limiting our ability to detect potential associations and weighting the analysis toward drugs in wide clinical use. Drotrecogin alfa had been withdrawn in 2011, nine years before the reporting period began. Conversely, this suggests that classical, widely used drugs may have higher detection sensitivity due to larger volumes of available data. The association reported for megestrol rests on 24 reports and its upper confidence limit lies close to 1, and should therefore be regarded as borderline. Furthermore, inferring causal relationships from observed associations remains challenging, and the influence of confounding factors cannot be completely eliminated. The direction of a reporting association also depends on which patients receive a drug. Indication bias and confounding by disease severity are therefore inherent to this design, as is the influence of concomitant medications, which cannot be separated from the drug of interest in spontaneous reports. Within the carbapenem class, the direction of association was not consistent, indicating that these associations are not explained by pharmacological class alone. This may reflect differences in the clinical settings in which the agents are used. Cilastatin is marketed only in fixed-dose combination with imipenem, and the two components cannot be separated in FAERS. One of the two Anatomical Therapeutic Chemical (ATC) codes used to identify cilastatin exposure (J01DH56; Table S11) corresponds to a triple combination that also contains relebactam, a β-lactamase inhibitor, so reports captured under this code cannot be attributed to cilastatin, imipenem, or relebactam individually. Relebactam is not marketed as a single agent, so it could not be examined as an independent comparator among the β-lactamase inhibitors (Figure S5C,D). The outcome was defined at the level of the “Coronavirus infections” high-level term, so the association is with that term group as a whole, and acute COVID-19 cannot be distinguished from post-acute, congenital, vaccine-derived or breakthrough presentations. Establishing any effect would require an interventional trial.

*Limitations of phenome analysis:* The LINCS database used is primarily based on drug responses in cancer cell lines, which exhibit different characteristics from alveolar epithelial cells, vascular endothelial cells, and immune cells, which are important in COVID-19 pathogenesis. Additionally, drug responses under the inflammatory environment in patients during SARS-CoV-2 infection may differ significantly from normal cell culture conditions. Furthermore, it is difficult to capture host–virus interactions and other complex biological processes that can only be detected in in vivo conditions using cell line-based phenome data. While extensive animal data have been accumulated and could potentially be curated and integrated into such databases, comprehensive incorporation remains challenging due to species differences in drug target molecules, including variations in binding site homology and pharmacological responses between species.

*Need for experimental validation:* This study primarily focused on computational predictions and analysis of existing databases to systematically identify therapeutic candidates and generate mechanistic hypotheses. While this approach provided valuable insights into drug repurposing opportunities for COVID-19 treatment, future studies should include direct validation using SARS-CoV-2-infected models and/or clinical evaluation. Such validation includes antiviral assays in infected respiratory cell systems, with target-engagement and rescue experiments as controls, and measurement of inflammatory cytokines. Animal studies in a SARS-CoV-2-susceptible model are also required before clinical evaluation.

## 4. Materials and Methods

### 4.1. Graph Attention Autoencoder (GATE)

#### 4.1.1. Knowledge Graph Construction

We constructed a heterogeneous knowledge graph comprising three node types—disease, drug, and gene (target)—with data primarily obtained from Open Targets (version 24.06) [20], which aggregates target–disease association data from multiple public sources. Four edge types were defined: (i) “Molecular interactions” (target–target), (ii) “Associations—direct (overall score)” (target–disease), (iii) “Drug—mechanism of action” (drug–target), and (iv) “Drug—indications” (drug–disease). The resulting knowledge graph comprised 11,882 disease, 4314 drug, and 11,448 gene nodes connected by 341,911 edges.

Each node was represented by a textual description of that node, and these descriptions were embedded into 768-dimensional vectors, which served as the initial node attributes of the model. The descriptions and their embeddings were produced by our proprietary LLM-based feature-engineering pipeline.

#### 4.1.2. Model Architecture and Training

Node embeddings were learned with a graph attention autoencoder (GATE) implemented in Python 3.10 using the Spektral library (v1.3.1) [55] with the TensorFlow 2.15.0/Keras backend. The encoder applied a single graph attention layer (GATConv) with 8 attention heads (concatenated; 256 channels per head) and an exponential linear unit (ELU) activation, with L2 regularization (2.5 × 10^−4^) applied to the kernel, attention-kernel, and bias weights. The decoder reconstructed the input node features through two fully connected layers (256 units with ReLU activation, followed by a linear layer matching the input feature dimension). The adjacency matrix was normalized using the standard GATConv preprocessing. The model was trained as a feature-reconstruction autoencoder by minimizing the mean squared error between the input and reconstructed node features (plus the L2 penalty), using the Adam optimizer (learning rate 1 × 10^−3^) in full-graph mode for approximately 4 × 10^4^ epochs, without dropout or early stopping. Negative sampling was not used, as the training objective was reconstruction of node features rather than prediction of edges. No validation split was used either: the objective is unsupervised reconstruction on a single fixed graph and every node is present during training, so no validation signal exists on which to select a checkpoint. A fixed random seed was set.

#### 4.1.3. Candidate Identification

With a single graph attention layer, each node representation is computed from the node’s own features and those of its immediate neighbors (its one-hop neighborhood), and from nothing else. Nodes whose neighborhoods contain similar information lie close in the latent space, even when the nodes are of different types, and drugs can be retrieved for a disease query in the absence of a direct edge. From the trained model, an attention-based latent node representation was used to rank all nodes by their cosine similarity to the COVID-19 node, with higher cosine similarity indicating greater proximity in the latent space. Drug nodes that fell within the top 500 of all ranked nodes were retained as candidates; different salt or derivative forms of the same compound (e.g., cilastatin and cilastatin sodium) were treated as a single drug entity. Candidates were thus defined by a rank-based cutoff: the top-500 cutoff was set in advance, no threshold was applied to the cosine similarity value itself, and cosine similarity was the only ranking metric used. The sensitivity of the retrieval result to the choice of cutoff is provided in Table S4. Each drug entity was then classified as having an established or a minimal prior association with COVID-19. An established association was defined as documented or otherwise recognized use of the drug in the clinical management of COVID-19 or its symptoms; this was assessed by searching the published literature and publicly available web sources for each drug. The drug entities with a minimal prior association were carried forward into the disproportionality and pathway profiling analyses. This search was not a systematic review.

#### 4.1.4. Retrieval Evaluation of the GATE Representation

To evaluate the latent space, we measured retrieval against repoDB. repoDB indications were mapped onto the knowledge graph by identifier: of the 6677 approved indications recorded in repoDB, 3107 could be mapped onto both a drug and a disease node identifier, and of these, 1404 had both nodes present in the graph, corresponding to 1393 unique drug–disease pairs. Of these pairs, the 586 that are present as training edges were used only to quantify memorization, and the remaining 807, which the graph does not contain as an edge, were used as positives (the unlinked indications); they span 239 diseases, which do not include COVID-19. No edge was removed from the graph for this evaluation, and no train–test split was constructed. Each disease was queried as in the candidate selection, ranking all 27,644 nodes by cosine similarity and, separately, the 4314 drug nodes alone. The ranking over all nodes was left unmodified, as in the candidate selection; on the drug-node ranking, drugs already linked to that disease by a training edge were removed, so that known indications do not fill the top of the list. Recall@N, the enrichment factor, and the median percentile rank were recorded on both axes. The same procedure was applied to the COVID-19 query, for which no unlinked indications exist, for 17 drugs authorized for COVID-19, recommended in guidelines, or evaluated in randomized trials in COVID-19.

Pairwise discrimination was assessed under three definitions of the negative set: the terminated, withdrawn, and suspended indications recorded in repoDB (*n* = 730), referred to as clinically hard negatives; degree-matched negatives; and random negatives, the latter two at ratios of 1:1 and 1:100, and each resampled five times. Four references were evaluated on the identical task: node2vec on the same graph (*p* = q = 1, 256 dimensions, 10 walks of length 80 per node, window 10, five epochs); the input text embedding, that is, the node descriptions before the graph encoder, which isolates the contribution of the encoder; common neighbors, a standard local-similarity measure; and preferential attachment, which ignores the query disease and ranks by degree alone, as a degree control. Degree was taken within the drug–disease edge set when ranking drugs and as total graph degree when ranking all nodes. Cutoffs from the top 100 to the top 10,000 nodes were examined.

To examine whether COVID-19-specific information had leaked into the model inputs, we analyzed the edges of the COVID-19 node (MONDO_0100096), the content of the node descriptions, and the composition of its one-hop neighborhood. Node descriptions were searched case-insensitively for COVID, SARS-CoV-2, and 2019-nCoV across all 27,633 nodes. Neighbors shared between the COVID-19 node and each retrieved drug node were counted and compared with the base rate across all 4314 drug nodes.

### 4.2. Disproportionality Analysis with Spontaneous Reporting Systems

We utilized a spontaneous adverse event (AE) reporting database constructed by the U.S. Food and Drug Administration (FDA). The FDA Adverse Event Reporting System (FAERS) contains reports of spontaneous AEs reported by consumers, pharmaceutical companies, and medical professionals worldwide. FAERS consists of seven datasets: DEMO (patient demographic and administrative information), DRUG (drug information), REAC (AEs), INDI (indications for the use of reported drugs), THER (start and end dates for the reported drugs), OUTC (outcomes for the event), and RPSR (sources for the event). The present study used FAERS data from January 2020 to March 2022, representing 3,617,885 reports after deduplication. These data were obtained through JAPIC-AERS (Japan Pharmaceutical Information Center (JAPIC), Tokyo, Japan), a commercially processed FAERS database. Duplicate reports in FAERS were identified and removed using CASEID, FDA_DT, and PRIMARYID. When duplicate CASEIDs were identified, the report with the latest FDA_DT was retained. In cases where both CASEID and FDA_DT were identical, the record with the largest PRIMARYID was selected.

AEs in the FAERS database are coded using Medical Dictionary for Regulatory Activities (MedDRA) Preferred Terms (PTs); the dictionary version used was MedDRA/J Ver. 27.1 J. COVID-19-related AE terms were defined as the preferred terms grouped under the MedDRA High Level Term (HLT) “Coronavirus infections” (code 10084510); the complete list of these 17 preferred terms is provided in Table S12, and this set is referred to as “coronavirus infections” in the figures. Drugs were identified by active ingredient, and fixed-dose combination products were not reconstructed from their component ingredients. The ATC codes used to identify each candidate drug are listed in Table S11. No restriction was applied by reported drug role; primary suspect, secondary suspect, concomitant, and interacting reports were all counted.

AE risk was evaluated by calculating the reporting odds ratio (ROR) and corresponding 95% confidence intervals (CI). The ROR and 95% CI were defined as follows: ROR = (a/c)/(b/d); 95% CI = exp [ln(ROR) ± 1.96√(1/a + 1/b + 1/c + 1/d)], where a, b, c, and d refer to the number of individuals in the FAERS database divided into the following: (a) those who received the drug of interest (e.g., cilastatin) and had COVID-19-related AE terms reported; (b) those who received the drug of interest without COVID-19-related AE terms; (c) those who did not receive the drug of interest and had COVID-19-related AE terms reported; and (d) those who did not receive the drug of interest and did not have COVID-19-related AE terms. If the upper limit of the 95% CI was <1, a significant inverse association was assumed between use of the drug of interest and the reporting of COVID-19-related AE terms.

### 4.3. Pathway Profiling

To explore potential therapeutic agents at the phenome level, we conducted analyses using pathway alteration profiles following drug treatment. We utilized gene expression profile data from the Expanded CMAP LINCS Resource 2020 [22], which contains transcriptome data generated from cell lines treated with various compounds. To mitigate noise inherent in high-throughput methodologies, we employed a quality control process based on gene expression distribution across replicate samples, retaining only high-confidence data; for each perturbation condition, approximately three replicate samples were aggregated into a single expression profile. Plate-to-plate normalization was performed to eliminate assay bias. Furthermore, we applied distribution analysis to determine gene expression alteration levels, selecting only profiles with gene alterations at a Q-value threshold of <0.20. Gene Set Enrichment Analysis (GSEA) [56,57] was conducted on the filtered profiles to identify significantly altered pathways for each profile, using a false discovery rate (FDR) cutoff of <0.20, with up- and down-regulated pathways considered separately. The GSEA output consists of normalized enrichment scores (NES) and FDR values estimated by permutation; confidence intervals are not produced by this analysis. For the GSEA analysis, we utilized ontology gene sets (C5) [58] from the Molecular Signatures Database (MSigDB) [59,60]. Correlations between pathway profiles were evaluated using Fisher’s exact test to identify compounds with similar phenotypic effects. The GSEA and the statistical analysis were implemented in house in C (compiled with GCC 11.3) and Ruby 3.2. Multiple testing correction was applied at the profile level using the Benjamini–Hochberg method, with an FDR threshold of 0.20 to determine statistical significance. These three thresholds, applied to profile selection, gene set significance, and profile-level correlation, were specified in advance given the exploratory, hypothesis-generating nature of the analysis. Pathway enrichment visualization was performed using Enrichment Map (v3.4.0) [61], a plugin for Cytoscape software (Version 3.10.1) [62]. Significantly enriched pathways with FDR < 0.20 were included in the analysis. The pathway network was constructed with each node representing an enriched gene set, and edges connecting pathways with shared genes, where the edge weight corresponds to the overlap coefficient between the gene sets. To improve interpretability of the resulting network, we utilized the AutoAnnotate plugin (v1.5.1) [63] to cluster and label groups of functionally related pathways based on their shared gene content. Clusters were manually reviewed and annotated to ensure biological relevance.

## 5. Conclusions

In this study, we systematically evaluated the applicability of existing drugs for COVID-19 treatment using the GATE-based latent space analysis with real-world evidence and phenome-level pathway profiling. The GATE-based analysis identified 16 candidate drug entities, 12 of which are already used or recognized in the context of COVID-19 or its symptoms; of the remaining four, the multilayered evaluation prioritized two for further investigation, where the real-world and phenome-level evidence converged: cilastatin and megestrol, both clinically approved medications whose documented safety profiles would need to be weighed against the risks specific to each drug.

Our multilayered evaluation approach supported candidate drug prioritization by assessing clinical and molecular biological plausibility. Integration of these heterogeneous data sources—static knowledge graphs, real-world clinical observations, and dynamic gene expression data—enabled multi-dimensional insights difficult to obtain with a single analytical method. Ranking by proximity in the GATE latent space is a deliberately permissive first phase and retrieves many drugs for which the available information is limited. Real-world data analysis indicated potential clinical relevance at the reporting level, while phenome data analysis supported molecular biological plausibility and mechanistic hypotheses for both drugs.

This integrated framework offers a practical approach to computational drug discovery aimed at narrowing the gap between in silico prediction and clinical translation. This multimodal methodology has applications for rapid candidate identification during pandemics and for diseases and conditions with limited therapeutic options. Future developments in large language models (LLMs) and agentic AI may further strengthen this multilayered framework, including by improving knowledge-graph comprehensiveness.

Future research should focus on in vivo validation and clinical trials of these repurposed drug candidates. It should also explore the applicability of this methodology to other diseases and establish selection criteria for optimal computational methods based on data scale and intended use.

## Figures and Tables

**Figure 1:**
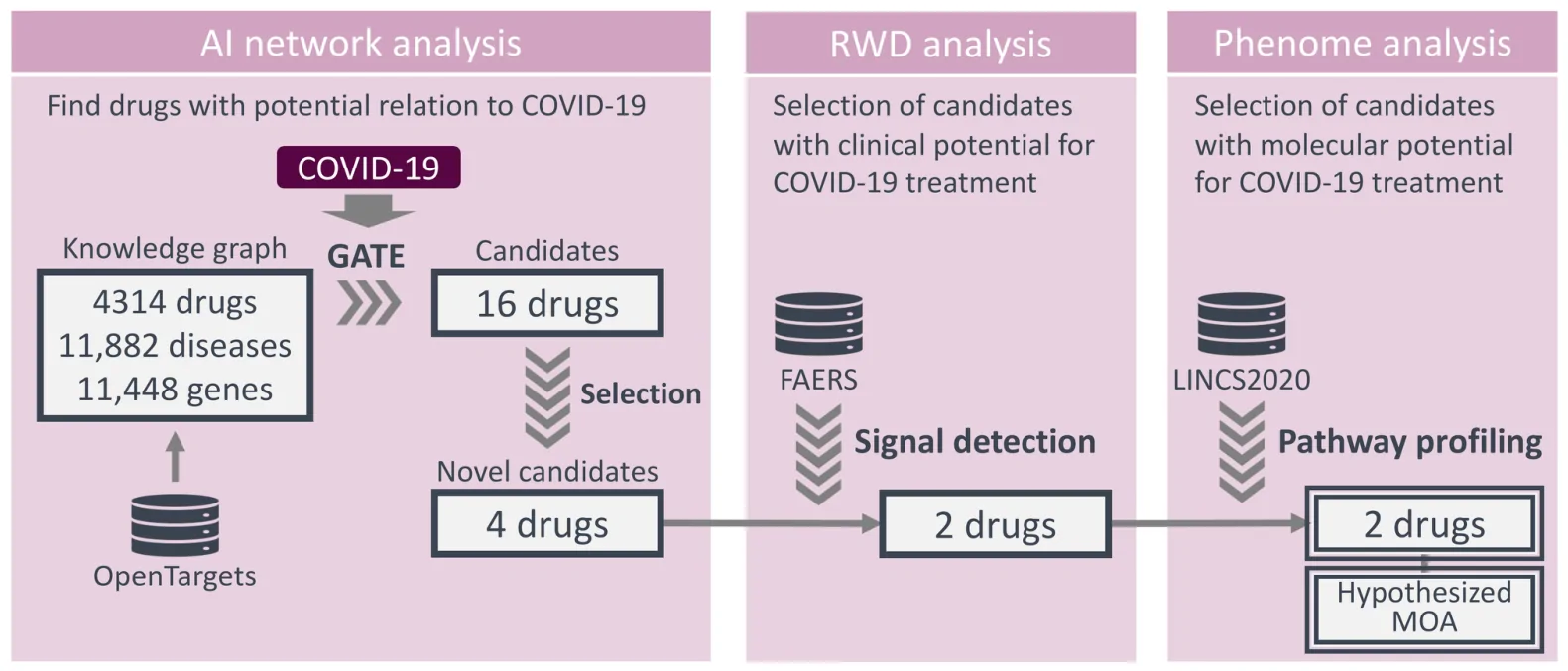
Workflow of analytical framework for COVID-19. AI network analysis using a graph attention autoencoder (GATE) selects candidate drugs for COVID-19, real-world data analysis (disproportionality signal detection) narrows them to drugs with inverse reporting signals for COVID-19-related adverse event (AE) terms, and phenome analysis (pathway profiling) evaluates their molecular plausibility and yields hypothesized mechanisms of action.

**Figure 2:**
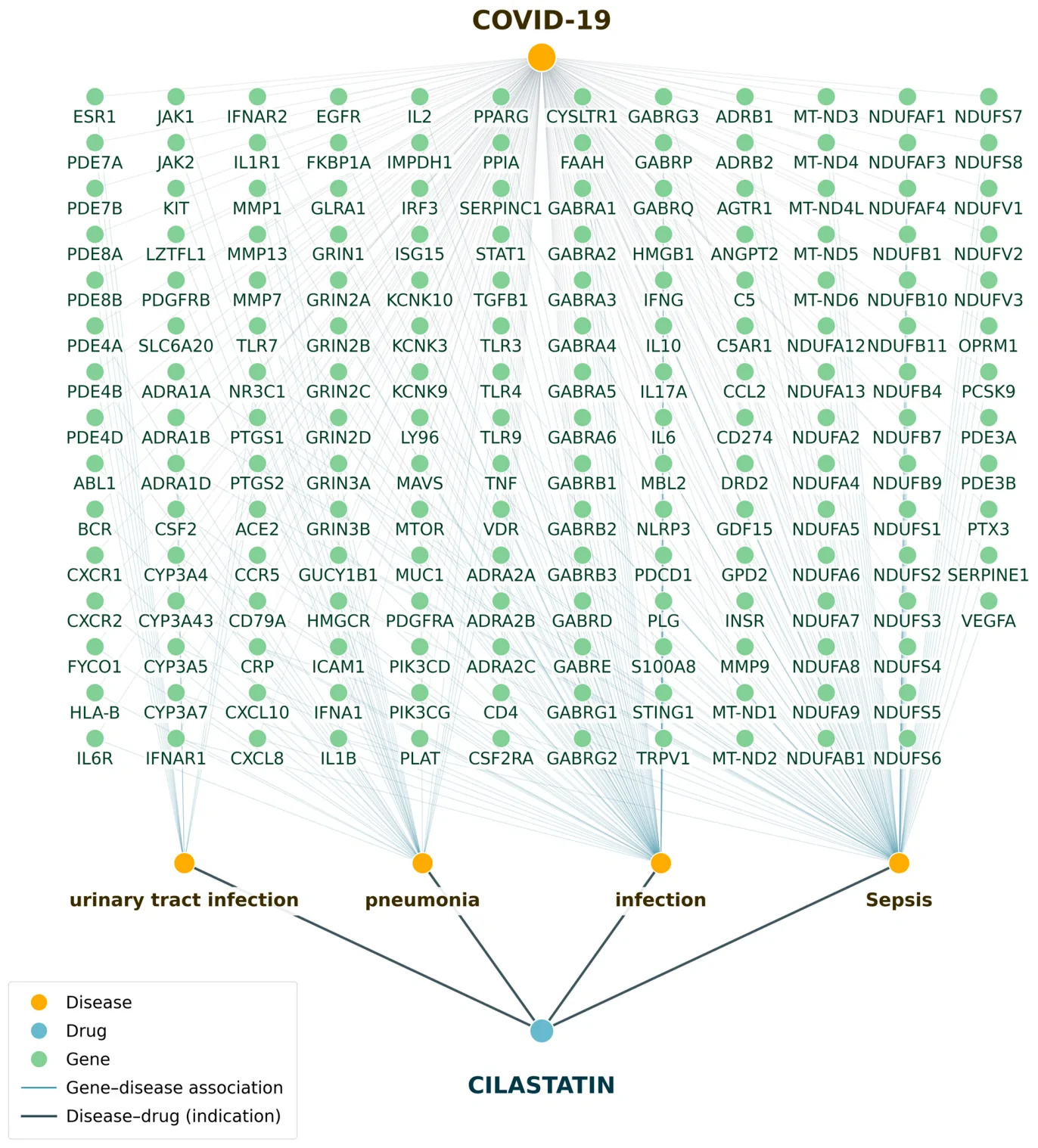
Subnetwork connecting COVID-19 and cilastatin in multimodal knowledge graph. The subnetwork comprises the nodes on the shortest paths between the COVID-19 and cilastatin nodes; the two share no direct edge and are linked indirectly through 177 genes shared with four cilastatin-indicated diseases (urinary tract infection, pneumonia, infection, and sepsis). Nodes are colored by type: orange for diseases, blue for drugs, and green for genes.

**Figure 3:**
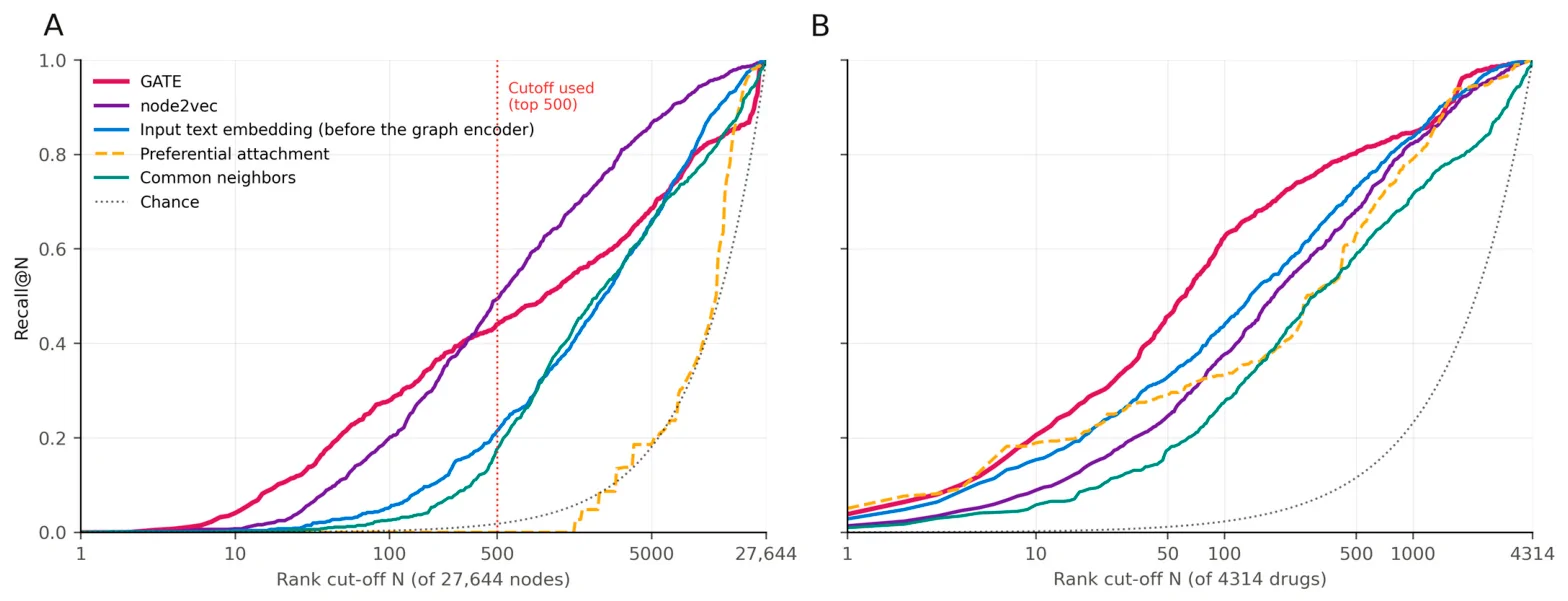
Retrieval of unlinked approved indications on two ranking axes. Recall@N is plotted against the rank cutoff N, for the 807 approved drug–disease indications in repoDB that span 239 diseases and that carry no corresponding edge in the graph. (**A**) Ranking over all 27,644 nodes of the graph; the vertical dotted line marks the top-500 cutoff used in this study. (**B**) Ranking over the 4314 drug nodes alone; the cutoff applies to the ranking in (**A**) and is not marked here. The GATE representation is compared with a node2vec embedding trained on the same graph, with the input text embedding from which the GATE latent space is derived, and with two graph-based baselines, common neighbors and preferential attachment; the dotted diagonal is the expectation under random ranking. Values at the cutoff are given in Table S1.

**Figure 4:**
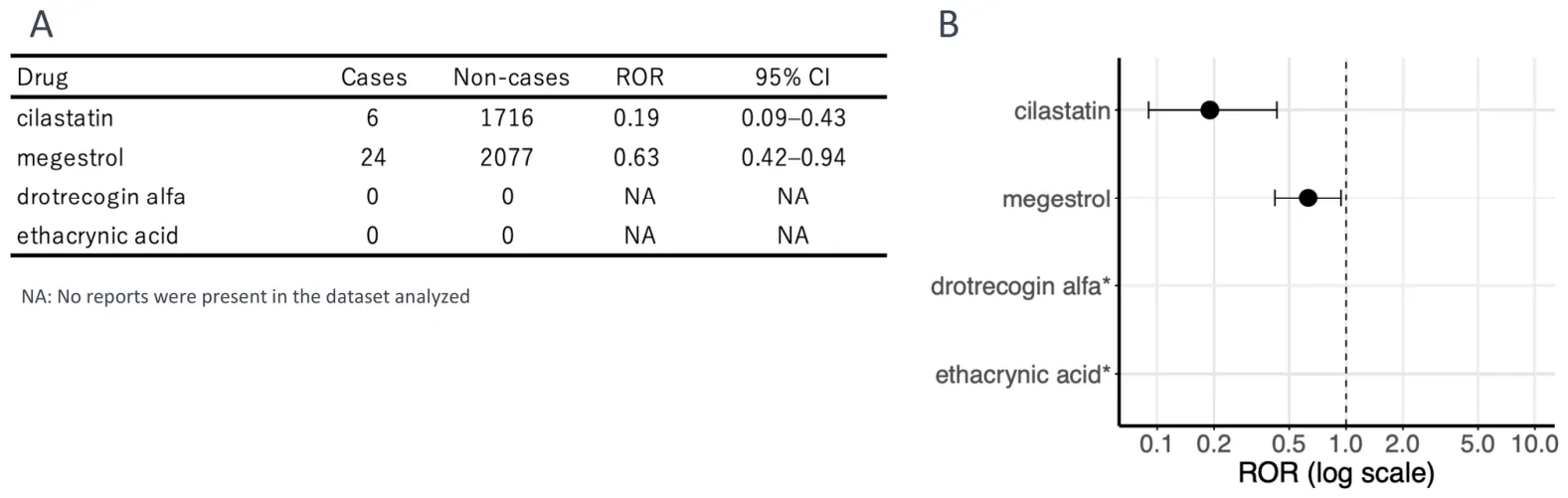
DPA of repurposing candidate drugs and coronavirus infections. (**A**) Case and non-case counts, reporting odds ratios (ROR) and 95% confidence intervals (CI) for candidate drugs through GATE-based analysis. Cilastatin is marketed only as a fixed-dose combination with imipenem, and the two components cannot be separated in these data. “NA” indicates that no reports for the drug were present in the dataset analyzed and that the ROR could not be calculated. (**B**) Forest plot visualizing RORs for the same set of drugs. The horizontal bars represent 95% CIs. RORs for drotrecogin alfa and ethacrynic acid were not calculable and are indicated with asterisks (*). Complete 2 × 2 counts for these drugs are given in Table S7.

**Figure 5:**
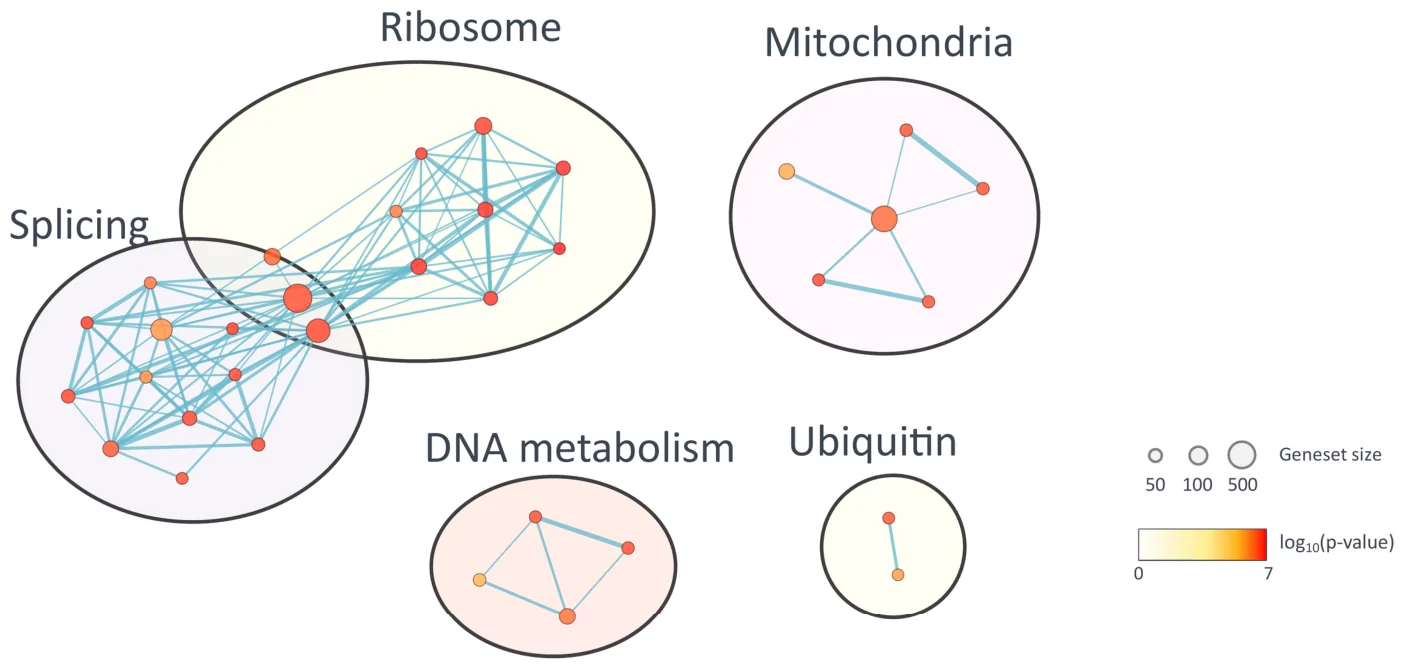
Enrichment map of down-regulated pathways following cilastatin treatment. This network visualizes gene sets from the MSigDB C5 collection significantly down-regulated by cilastatin treatment. Each node represents a gene set, with node size proportional to the number of genes in the set and node color reflecting statistical significance (log_10_-transformed *p*-value). Edges indicate gene overlap between gene sets. Functionally related clusters are annotated.

**Figure 6:**
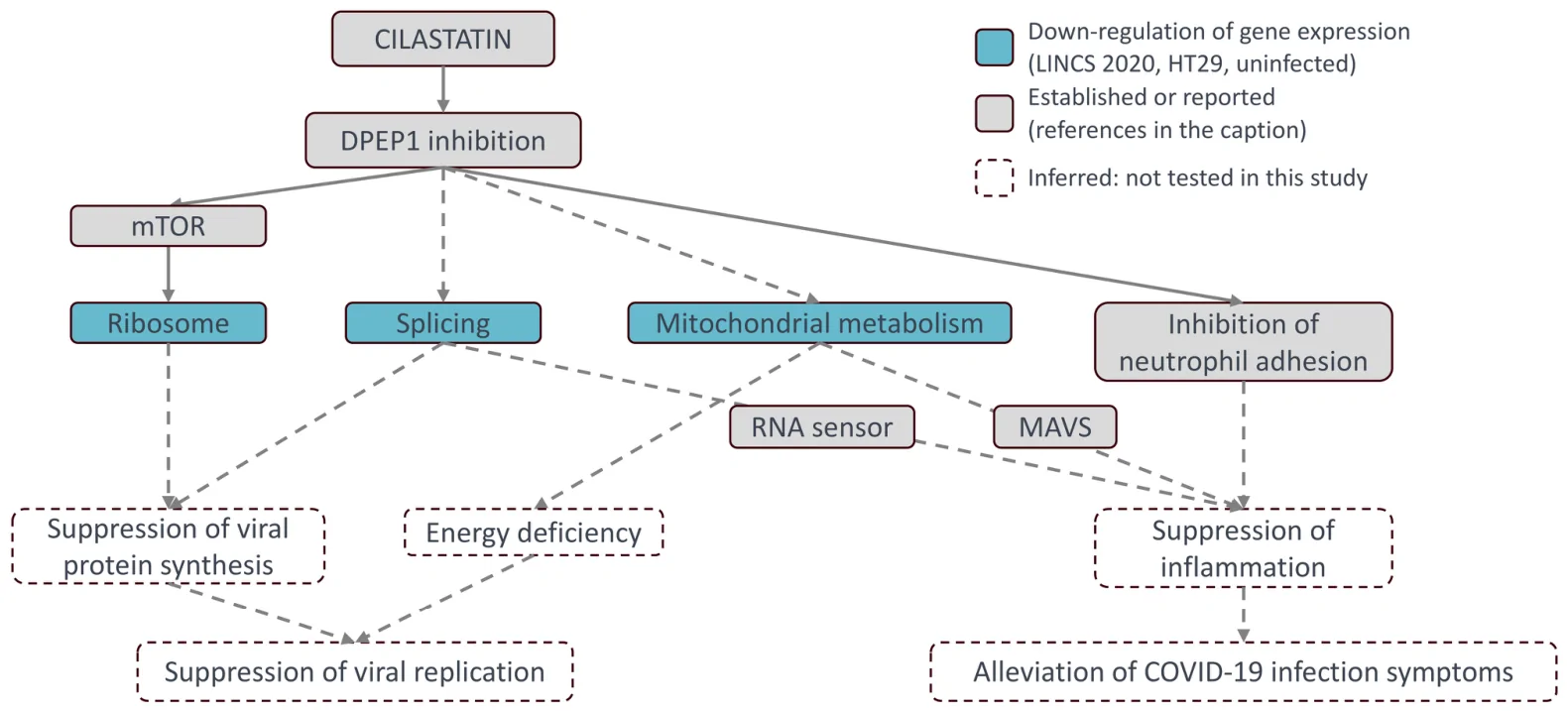
Hypothesized mechanisms of action of cilastatin in COVID-19. This schematic illustrates potential therapeutic mechanisms by which cilastatin may alleviate COVID-19 pathology, inferred from pathway-level gene expression changes. Cilastatin-mediated inhibition of DPEP1 is proposed to influence multiple downstream biological processes, including ribosome biogenesis, RNA splicing, and mitochondrial metabolism. These changes converge on antiviral and anti-inflammatory outcomes such as suppression of viral replication and inflammation. Pathways shaded in blue indicate those with down-regulated gene expression following cilastatin treatment in uninfected HT29 cells (LINCS 2020). Elements shaded in grey are established or are supported by the cited references: inhibition of neutrophil adhesion [48], mTOR [49], RNA sensor [50], and MAVS [51]. Elements and links drawn with dashed lines were inferred and were not tested in this study; neither target engagement nor rescue was assessed. This model is predictive and awaits experimental confirmation.

**Table 1:** Top-ranked drug candidates based on embedding similarity to COVID-19 in the latent space learned by the GATE model.

ID	Name	Description	Cosine Similarity	OpenTargets Association Score	OpenTargets Rank of Score
**CHEMBL1201057**	**CILASTATIN SODIUM**	**Renal dehydropeptidase-I (DPEP1) inhibitor**	**0.86067**	**0.01**	**2672**
**CHEMBL766**	**CILASTATIN**	**Renal dehydropeptidase-I (DPEP1) inhibitor**	**0.86006**	**0.01**	**2672**
CHEMBL1201014	PREDNISOLONE SODIUM PHOSPHATE	Steroid Drug	0.85693	0.609	4
CHEMBL1201231	PREDNISOLONE PHOSPHORIC ACID	Steroid Drug	0.85526	0.609	4
CHEMBL679	EPINEPHRINE	Adrenergic Receptor Agonist	0.85111	0.372	169
CHEMBL2134724	IPRATROPIUM BROMIDE	Anticholinergic Drug, COPD and Bronchial Asthma Treatment	0.84696	0.076	737
CHEMBL1621597	IPRATROPIUM	Anticholinergic Drug, COPD and Bronchial Asthma Treatment	0.84621	0.076	737
CHEMBL1200689	NITRIC OXIDE	Nitric Oxide, Pulmonary Hypertension Treatment	0.84535	0.345	397
CHEMBL1201335	GLYCOPYRRONIUM	Anticholinergic Drug	0.84439	0.076	737
CHEMBL1437	NOREPINEPHRINE	Adrenergic Receptor Agonist	0.84266	0.372	169
CHEMBL1215	PHENYLEPHRINE	Adrenergic Receptor Agonist	0.84121	0.372	169
CHEMBL2108429	MEPOLIZUMAB	Anti-IL-5 Antibody	0.83772	0.068	799
**CHEMBL1201139**	**MEGESTROL ACETATE**	**Antineoplastic Drug, Contraceptive, Progesterone Receptor Agonist**	**0.83667**	**0.086**	**636**
CHEMBL1370	BUDESONIDE	Steroid Drug	0.83639	0.609	4
CHEMBL1364144	METHYLPREDNISOLONE ACETATE	Steroid Drug	0.83551	0.609	4
CHEMBL1201027	GLYCOPYRROLATE	Anticholinergic Drug	0.83493	0.076	737
**CHEMBL2109065**	**DROTRECOGIN ALFA (ACTIVATED)**	**Antithrombotic, Anti-inflammatory, Fibrinolysis-promoting Human Activated Protein C**	**0.83448**	**0.026**	**1339**
CHEMBL52440	DEXTROMETHORPHAN	Centrally acting antitussive	0.83335	0.365	334
CHEMBL1473	FLUTICASONE PROPIONATE	Steroid Drug	0.83256	0.609	4
CHEMBL3707243	GLYCOPYRRONIUM TOSYLATE	Anticholinergic Drug	0.83239	0.076	737
**CHEMBL456**	**ETHACRYNIC ACID**	**Loop Diuretic Drug**	**0.83171**	**0.004**	**4822**

Drug candidates are ranked by cosine similarity scores in the GATE latent space. The table lists the 21 drug nodes that fell within the top 500 of all ranked nodes; because different salt or derivative forms of the same compound were treated as a single drug entity, these correspond to 16 distinct drug entities. Rows shown in bold indicate drugs not previously used for COVID-19 treatment, representing candidates with minimal prior COVID-19 associations. The OpenTargets association scores range from 0 to 1, with higher values indicating stronger relevance.

**Table 2:** Top-ranked compounds positively correlated with the pathway profile of cilastatin.

PertID	Name	MOA	Cell	Conc	Time	Adj. *p*-Value	Registered COVID-19 Trials	COVID-19 Reports
BRD-K79759031	artesunate	—	HT29	10 µM	6 h	4.4 × 10^−42^	10	—
BRD-K66896231	ipidacrine	Acetylcholinesterase inhibitor	HT29	10 µM	6 h	1.0 × 10^−40^	—	structural [23]
BRD-K60067222	BRD-K60067222	—	PC3	10 µM	24 h	8.7 × 10^−40^	—	—
BRD-K68185022	ursolic-acid	11-beta hydroxysteroid dehydrogenase inhibitor	PC3	70 µM	24 h	4.2 × 10^−39^	—	reviews [24,25], docking [26]
BRD-A19037878	BRD-A19037878	—	K562	2.5 µM	24 h	8.0 × 10^−39^	—	—
BRD-A75409952	wortmannin	PI3K inhibitor	PC3	10 µM	6 h	2.6 × 10^−38^	—	computational [27,28]
BRD-K81855038	roxatidine	—	HT29	10 µM	6 h	4.9 × 10^−38^	—	—
BRD-A79768653	sirolimus	MTOR inhibitor	PC3	3.33 µM	24 h	1.5 × 10^−37^	7	—
BRD-K69023402	thapsigargin	ATPase inhibitor	JURKAT	0.37 µM	24 h	1.5 × 10^−37^	—	experimental [29,30,31]
BRD-K56032964	AP-26113	—	HT29	0.37 µM	24 h	1.6 × 10^−37^	—	—

A dash in the MOA column indicates that no mechanism-of-action annotation is available in the LINCS 2020 metadata. The “Registered COVID-19 trials” and “COVID-19 reports” columns indicate previously reported associations with COVID-19. Report types are experimental (cell- or virus-based assays), structural (crystallographic binding to the main protease, without demonstrated antiviral activity), computational (network or machine-learning prediction), docking (molecular docking only), and review. A dash in these two columns indicates that none was identified. The status, phase, design, and intervention of each trial are given in Table S8.

**Table 3:** Top-ranked compounds positively correlated with the pathway profile of megestrol.

PertID	Name	MOA	Cell	Conc	Time	Adj. *p*-Value	Registered COVID-19 Trials	COVID-19 Reports
BRD-K37142460	MI-2	—	OCILY3	10 µM	24 h	1.3 × 10^−8^	—	—
BRD-A81370665	BI-D1870	Ribosomal protein inhibitor	HBL1	10 µM	24 h	1.3 × 10^−8^	—	—
BRD-K01436366	XMD-1150	Leucine rich repeat kinase inhibitor	BJAB	10 µM	24 h	3.4 × 10^−8^	—	—
BRD-K36363294	I-BET-151	—	HIMG002	2.5 µM	24 h	3.4 × 10^−8^	—	—
BRD-A53077924	tianeptine	Selective serotonin reuptake enhancer	HIMG001	15 µM	24 h	4.1 × 10^−8^	—	—
BRD-K12502280	TG-101348	FLT3 inhibitor	OCILY3	10 µM	4 h	2.5 × 10^−7^	—	review [32]
BRD-K58772419	AZD-6482	PI3K inhibitor	MCF7	1.11 µM	24 h	3.0 × 10^−7^	—	structural [23]
BRD-K12502280	TG-101348	FLT3 inhibitor	TMD8	2.5 µM	4 h	5.7 × 10^−7^	—	review [32]
BRD-K81418486	vorinostat	HDAC inhibitor	JURKAT	10 µM	24 h	5.7 × 10^−7^	—	experimental [33], computational [34,35]
BRD-K54606188	BRD-K54606188	—	TMD8	0.66 µM	4 h	1.9 × 10^−6^	—	—

A dash in the MOA column indicates that no mechanism-of-action annotation is available in the LINCS 2020 metadata. The “Registered COVID-19 trials” and “COVID-19 reports” columns indicate previously reported associations with COVID-19. Report types are experimental (cell- or virus-based assays), structural (crystallographic binding to the main protease, without demonstrated antiviral activity), computational (network or machine-learning prediction), docking (molecular docking only), and review. A dash in these two columns indicates that none was identified. The status, phase, design, and intervention of each trial are given in Table S8.

## Data Availability

The datasets presented in this article are not readily available because the spontaneous adverse event data were obtained from JAPIC-AERS, a commercially licensed database derived from the FDA Adverse Event Reporting System (FAERS), and the node features and analysis code are proprietary to GEXVal Inc., although the original contributions presented in this study are included in the article and the Supplementary Material. Requests to access the datasets should be directed to the corresponding author.

## Supplementary Materials

The following supporting information can be downloaded at: https://www.mdpi.com/article/10.3390/ph19101522/s1. Table S1: Retrieval of unlinked approved indications, ranked over all 27,644 nodes (A) and over the 4314 drug nodes alone (B); Table S2: Pairwise discrimination of individual drug–disease pairs under five negative sets, by AUROC (A) and AUPR (B), and by presence of a corresponding training edge (C); Table S3: Ranks of the 17 drugs used or evaluated in COVID-19 that are present in the graph, for the COVID-19 query and across 239 other disease queries; Table S4: Sensitivity of recovery and enrichment to the rank cutoff; Table S5: Gene neighborhood shared with the COVID-19 node, for the 21 drug nodes within the top 500 (A) and for the 17 drugs used or evaluated in COVID-19 (B); Table S6: Nodes whose description text mentions COVID-19 or SARS-CoV-2 (20 of 27,633); Table S7: Complete 2 × 2 counts and reporting odds ratios for the candidate drugs and for the comparator classes; Table S8: Registered COVID-19 clinical trials cited for the compounds in Table 2 and Table 3; Table S9: Significantly down-regulated gene sets in cilastatin-treated HT29 cells (LINCS 2020); Table S10: Disease queries that rank the 17 drugs used or evaluated in COVID-19 higher than the COVID-19 query does; Table S11: ATC codes used to identify the candidate drugs in the reporting database; Table S12: Preferred terms grouped under the MedDRA High Level Term “Coronavirus infections”; Figure S1: Subnetwork connecting COVID-19 and megestrol in multimodal knowledge graph; Figure S2: Subnetwork connecting COVID-19 and drotrecogin alfa in multimodal knowledge graph; Figure S3: Subnetwork connecting COVID-19 and ethacrynic acid in multimodal knowledge graph; Figure S4: Retrieval of unlinked approved indications by three models trained from independent initializations; Figure S5: DPA of carbapenems and of β-lactamase inhibitors, and coronavirus infections; Figure S6: DPA of progestins and related drug classes and coronavirus infections.
