# Supplementary material for: Multilayered Prioritization of Graph Attention Autoencoder–Derived Drug Repurposing Candidates for COVID-19
Source: Pharmaceuticals (Basel). 2026 Sep 25;19(10):1522. doi: 10.3390/ph19101522 (PMC13708601; doi:10.3390/ph19101522)
Supplement: Supplementary file 1 [file pharmaceuticals-19-01522-s001.zip › Supplementary Tables.pdf]

Supplementary Tables

Table S1 Retrieval of unlinked approved indications, ranked over all 27,644 nodes (A) and over the 4314 drug nodes alone (B)

A. Ranking over all 27,644 nodes

Representation	Recall@10	Recall@50	Recall@100	Recall@500	median percentile (%)
GATE	0.041	0.211	0.280	0.440	3.66
node2vec	0.007	0.113	0.200	0.496	1.87
Input text embedding	0.004	0.029	0.052	0.214	8.87
Preferential attachment	0.000	0.000	0.000	0.000	47.33
Common neighbors	0.001	0.011	0.026	0.178	8.02

B. Ranking over the 4314 drug nodes

Representation	Recall@10	Recall@50	Recall@100	Recall@500	median percentile (%)
GATE	0.206	0.456	0.627	0.803	1.41
node2vec	0.089	0.248	0.378	0.682	4.24
Input text embedding	0.154	0.332	0.437	0.730	3.18
Preferential attachment	0.190	0.292	0.333	0.634	6.37
Common neighbors	0.058	0.174	0.278	0.589	6.93

Note. Positives are the 807 approved drug–disease indications recorded in RepoDB across 239 diseases that carry no corresponding edge in the training graph (unlinked indications).

Recall@N is the proportion of unlinked indications whose drug falls within the first N nodes of the ranking for its own disease query.

Median percentile is the median rank of an unlinked indication expressed as a percentage of the nodes ranked; lower is better.

Table S2 Pairwise discrimination of individual drug–disease pairs under five negative sets, by AUROC (A) and AUPR (B), and by presence of a corresponding training edge (C)

A. AUROC

Representation	random 1:1	degree-matched 1:1	random 1:100	degree-matched 1:100	clinically hard 1:0.9
GATE	0.909 ± 0.003	0.742 ± 0.006	0.908 ± 0.000	0.744 ± 0.000	0.573
Input text embedding	0.914 ± 0.002	0.838 ± 0.008	0.915 ± 0.000	0.839 ± 0.001	0.701
Common neighbors	0.823 ± 0.004	0.773 ± 0.011	0.824 ± 0.000	0.774 ± 0.001	0.500
Preferential attachment	0.887 ± 0.006	0.556 ± 0.002	0.890 ± 0.000	0.555 ± 0.000	0.392

B. AUPR

Representation	random 1:1	degree-matched 1:1	random 1:100	degree-matched 1:100	clinically hard 1:0.9
GATE	0.921 ± 0.007	0.790 ± 0.010	0.293 ± 0.007	0.085 ± 0.001	0.586
Input text embedding	0.919 ± 0.003	0.851 ± 0.008	0.283 ± 0.011	0.129 ± 0.005	0.711
Common neighbors	0.814 ± 0.008	0.735 ± 0.018	0.198 ± 0.005	0.041 ± 0.001	0.519
Preferential attachment	0.868 ± 0.009	0.572 ± 0.004	0.102 ± 0.002	0.018 ± 0.001	0.459
Chance level	0.5	0.5	0.0099	0.0099	0.525

C. AUROC by presence of a corresponding training edge

Representation	in-graph (586 pairs)	unlinked (807 pairs)	Difference
GATE	0.965	0.909	0.056
Input text embedding	0.952	0.921	0.032

Note. In A and B, positives are the 807 approved drug–disease pairs recorded in RepoDB that carry no corresponding edge in the training graph (unlinked indications).

"random" negative sets draw drug–disease pairs that are absent from both the graph and RepoDB. In the "degree-matched" sets, each negative pair keeps the disease of the positive pair and replaces the drug with one of comparable degree: the 4314 drug nodes were ranked by degree and split into ten groups of equal size, and the replacement was drawn from the same group. A method that scored pairs by degree alone would therefore not separate positives from negatives. "clinically hard" uses the 730 indications recorded in RepoDB as terminated, withdrawn or suspended.

Values are the mean ± standard deviation over five independent negative samples; the clinically hard set is deterministic and was evaluated once.

AUPR depends on the proportion of positives, which differs between negative sets; the chance level of each set is given for comparison. AUROC has a chance level of 0.500 throughout.

In this table both graph-based baselines are computed over the whole graph without regard to edge type: common neighbors is the number of nodes adjacent to both the drug and the disease, and preferential attachment is the product of their degrees.

C reports AUROC after dividing the approved indications recorded in RepoDB by whether the graph contains a corresponding drug–disease edge: 586 are present as training edges and 807 are not. Negatives there are drawn 1:1 from pairs absent from both the graph and RepoDB in a single draw, so the unlinked values differ slightly from the five-draw means given in the random 1:1 column of A.

Table S3 Ranks of the 17 drugs used or evaluated in COVID-19 that are present in the graph, for the COVID-19 query and across 239 other disease queries

Drug	Class	ChEMBL identifier	Rank (all nodes)	Rank (drug nodes)	Percentile (drug nodes, %)	Within the top 500 (all nodes)	Median rank (other 239 queries)	Higher for COVID-19
Budesonide	inhaled corticosteroid	CHEMBL1370	361	14	0.32	yes	202	yes
Ritonavir	antiviral (Paxlovid component)	CHEMBL163	535	23	0.53	no	352	yes
Methylprednisolone	corticosteroid	CHEMBL650	784	34	0.79	no	79	yes
Dexamethasone	corticosteroid (RECOVERY)	CHEMBL384467	940	43	1.00	no	58	yes
Prednisolone	corticosteroid	CHEMBL131	950	44	1.02	no	57	yes
Hydrocortisone	corticosteroid	CHEMBL389621	1070	50	1.16	no	66	yes
Anakinra	IL-1 receptor antagonist	CHEMBL1201570	1836	71	1.65	no	237	yes
Tocilizumab	IL-6R antibody	CHEMBL1237022	2521	94	2.18	no	220	yes
Sarilumab	IL-6R antibody	CHEMBL2108730	4118	135	3.13	no	391	yes
Interferon beta-1a	interferon	CHEMBL1201562	5094	169	3.92	no	242	yes
Baricitinib	JAK inhibitor	CHEMBL2105759	6284	236	5.47	no	521	yes
Hydroxychloroquine	tested, no benefit	CHEMBL1535	7599	317	7.35	no	570	yes
Enoxaparin sodium	anticoagulant	CHEMBL1201476	8166	379	8.79	no	376	no
Tofacitinib	JAK inhibitor	CHEMBL221959	13822	853	19.77	no	985	yes
Colchicine	anti-inflammatory	CHEMBL107	24291	1648	38.20	no	1643	no
Vilobelimab	anti-C5a antibody	CHEMBL2109636	24949	1887	43.74	no	2186	yes
Heparin	anticoagulant	CHEMBL4650299	25448	2300	53.31	no	2020	no

Note. The first block gives two rankings: over all 27,644 nodes and over the 4314 drug nodes alone.

Percentile is the rank expressed as a percentage of the 4314 drug nodes.

These drugs were not used to fit or to select the model; they are reported as a positive control on the COVID-19 query.

The other 239 queries are the diseases of the RepoDB evaluation. For each query the 17 drugs were ranked among the 4314 drug nodes in the same way. The last two columns give the median of those 239 ranks for each drug, and whether the drug ranks higher for COVID-19 than for that median; 14 of the 17 do.

The queries placed above COVID-19 on that ordering are listed in Table S10.

Table S4 Sensitivity of recovery and enrichment to the rank cutoff

Cutoff N (of 27,644 nodes)	Unlinked approved indications recovered	Recall	Chance	Enrichment (fold)
100	226 of 807	0.28	0.0036	77.4169
250	309 of 807	0.3829	0.009	42.3395
500	355 of 807	0.4399	0.0181	24.3212
1000	401 of 807	0.4969	0.0362	13.7364
2000	460 of 807	0.57	0.0723	7.8787
3000	492 of 807	0.6097	0.1085	5.6179
5000	554 of 807	0.6865	0.1809	3.7955
10000	651 of 807	0.8067	0.3617	2.23

Note. The cutoff of 500 nodes used in this study is shown in bold. Recall is the proportion of the 807 unlinked approved indications in RepoDB, across 239 diseases, whose drug falls within the cutoff for its own disease query; chance is the cutoff expressed as a fraction of the 27,644 nodes; enrichment is their ratio.

Recovery and enrichment trade off continuously: widening the cutoff raises recall and lowers enrichment at every step.

This table is derived from RepoDB alone and is independent of the COVID-19 query. The ranks of the agents used or evaluated in COVID-19 are reported separately in Table S3.

Table S5 Gene neighborhood shared with the COVID-19 node, for the 21 drug nodes within the top 500 (A) and for the 17 drugs used or evaluated in COVID-19 (B)

A. The 21 drug nodes within the top 500 of all ranked nodes

Drug	Rank (all nodes)	Gene neighbors	Shared with COVID-19	Shared genes	Graph distance to COVID-19
Cilastatin sodium	50	1	0		3
Cilastatin	53	1	0		3
Prednisolone sodium phosphate	68	1	1	NR3C1	2
Prednisolone phosphoric acid	77	1	1	NR3C1	2
Epinephrine	95	8	8	ADRA1A;ADRA1B;ADRA1D;ADRA2A;ADRA2B;ADRA2C;ADRB1;ADRB2	2
Ipratropium bromide	139	1	0		3
Ipratropium	152	1	0		3
Nitric oxide	165	3	1	GUCY1B1	2
Glycopyrronium	170	5	0		3
Norepinephrine	203	8	8	ADRA1A;ADRA1B;ADRA1D;ADRA2A;ADRA2B;ADRA2C;ADRB1;ADRB2	2
Phenylephrine	233	3	3	ADRA1A;ADRA1B;ADRA1D	2
Mepolizumab	324	1	0		3
Megestrol acetate	357	1	0		3
Budesonide	361	1	1	NR3C1	2
Methylprednisolone acetate	386	1	1	NR3C1	2
Glycopyrrolate	398	2	0		3
Drotrecogin alfa (activated)	412	2	0		3
Dextromethorphan	439	2	1	GRIN2A	2
Fluticasone propionate	467	1	1	NR3C1	2
Glycopyrronium tosylate	475	5	0		3
Ethacrynic acid	492	1	0		3

B. The 17 drugs used or evaluated in COVID-19 that are present in the graph

Drug	Rank (drug nodes)	Gene neighbors	Shared with COVID-19	Shared genes
Budesonide	14	13	1	NR3C1
Ritonavir	23	9	4	CYP3A4;CYP3A43;CYP3A5;CYP3A7
Methylprednisolone	34	18	1	NR3C1
Dexamethasone	43	80	1	NR3C1
Prednisolone	44	59	1	NR3C1
Hydrocortisone	50	36	1	NR3C1
Anakinra	71	5	1	IL1R1
Tocilizumab	94	7	1	IL6R
Sarilumab	135	3	1	IL6R
Interferon beta-1a	169	4	2	IFNAR1;IFNAR2
Baricitinib	236	4	2	JAK1;JAK2
Hydroxychloroquine	317	4	2	TLR7;TLR9
Enoxaparin sodium	379	9	1	SERPINC1
Tofacitinib	853	6	4	JAK1;JAK2;JAK3;TYK2
Colchicine	1648	17	9	TUBB;TUBB1;TUBB2A;TUBB2B;TUBB3;TUBB4A;TUBB4B;TUBB6;TUBB8
Vilobelimab	1887	1	1	C5
Heparin	2300	1	1	SERPINC1

Note. "Shared with COVID-19" is the number of the 462 gene neighbors of the COVID-19 node that are also neighbors of the drug. Graph distance is the length of the shortest path to the COVID-19 node.

Ranks in A are over all 27,644 nodes; ranks in B are over the 4314 drug nodes.

All 17 drugs in B share at least one of the 462 genes. For most of them the shared gene is a drug target; for ritonavir the shared genes are cytochrome P450 3A enzymes, which are metabolizing enzymes rather than targets.

Table S6 Nodes whose description text mentions COVID-19 or SARS-CoV-2 (20 of 27,633)

Node type	Identifier	Name	Matched term(s)
drug	CHEMBL4297680	1018 ISS	COVID
drug	CHEMBL2393130	Apabetalone	COVID
drug	CHEMBL4297502	Avoralstat	COVID; SARS-CoV-2
drug	CHEMBL2165191	AZD-6482	COVID; SARS-CoV-2
drug	CHEMBL3707325	Lenzilumab	COVID
drug	CHEMBL4297905	Osocimab	COVID
drug	CHEMBL4650402	Posdinemab	COVID; SARS-CoV-2
drug	CHEMBL4594258	Selgantolimod	COVID; SARS-CoV-2
drug	CHEMBL2108167	Vepalimomab	COVID
drug	CHEMBL2424780	Vesatolimod	COVID
drug	CHEMBL3707293	XEN-D0501	COVID
drug	CHEMBL4650486	Zansecimab	COVID; SARS-CoV-2
disease	MONDO_0100096	COVID-19	COVID; SARS-CoV-2
disease	HP_0100806	Sepsis	COVID
disease	EFO_0007224	coronavirus infectious disease	COVID; SARS-CoV-2
disease	MONDO_0026767	immunodeficiency 74, COVID-19-related, X-linked	COVID
disease	EFO_0007956	monocyte:lymphocyte ratio	COVID
gene	ENSG00000130234	ACE2	SARS-CoV-2
gene	ENSG00000134545	KLRC1	COVID
gene	ENSG00000184012	TMPRSS2	SARS-CoV-2

Note. The node descriptions are the text from which the initial node features were generated; the search also allowed optional hyphens in the strings.
Twenty of the 27,633 nodes carrying a description (0.07%) matched. The description text itself is not reproduced here.
Matches on the broader terms coronavirus, pandemic, SARS and MERS are not listed, since these also occur in contexts unrelated to COVID-19.

Table S7 Complete 2 × 2 counts and reporting odds ratios for the candidate drugs and for the comparator classes

Series	Drug	(a) Drug and coronavirus term	(b) Drug, no such term	(c) No drug, coronavirus term	(d) Neither	ROR	95% CI
Candidate drugs	Cilastatin	6	1716	65,119	3,551,044	0.19	0.09–0.43
Candidate drugs	Megestrol	24	2077	65,101	3,550,683	0.63	0.42–0.94
Candidate drugs	Drotrecogin alfa (activated)	0	0	65,125	3,552,760	not calculable	
Candidate drugs	Ethacrynic acid	0	0	65,125	3,552,760	not calculable	
Carbapenems	Imipenem	14	2422	65,111	3,550,338	0.32	0.19–0.53
Carbapenems	Meropenem	235	8096	64,890	3,544,664	1.59	1.39–1.81
Carbapenems	Ertapenem	11	1578	65,114	3,551,182	0.38	0.21–0.69
Carbapenems	Doripenem	4	126	65,121	3,552,634	1.73	0.64–4.69
Carbapenems	Biapenem	2	59	65,123	3,552,701	1.85	0.45–7.57
β-Lactamase inhibitors	Clavulanic acid	71	4462	65,054	3,548,298	0.87	0.69–1.10
β-Lactamase inhibitors	Sulbactam	34	1720	65,091	3,551,040	1.08	0.77–1.51
β-Lactamase inhibitors	Tazobactam	287	12,031	64,838	3,540,729	1.30	1.16–1.46
β-Lactamase inhibitors	Avibactam	15	584	65,110	3,552,176	1.40	0.84–2.34
β-Lactamase inhibitors	Sultamicillin	3	186	65,122	3,552,574	0.88	0.28–2.75
Progesterone-receptor agonists and related classes	Sex hormones	332	34,697	64,793	3,518,063	0.52	0.47–0.58
Progesterone-receptor agonists and related classes	Anticancer agents	52	4594	65,073	3,548,166	0.62	0.47–0.81
Progesterone-receptor agonists and related classes	Systemic hormonal contraceptives	283	31,764	64,842	3,520,996	0.48	0.43–0.54
Progesterone-receptor agonists and related classes	Progesterone	117	19,510	65,008	3,533,250	0.33	0.27–0.39

Note. Marginal totals are the 3,617,885 reports retained after deduplication for the period analyzed and the 65,125 of these that contained a preferred term in the "Coronavirus infections" high level term.

Drotrecogin alfa and ethacrynic acid have no report in the period analyzed, so no ratio can be calculated; drotrecogin alfa was withdrawn in 2011. Cilastatin is marketed only as a fixed-dose combination with imipenem, and the two components cannot be separated in these data.

Reporting odds ratios for the candidate drugs are shown in Figure 4, those for the comparator series in Figure S5 (carbapenems and β-lactamase inhibitors, for cilastatin) and Figure S6 (progesterone-receptor agonists and related classes, for megestrol); the comparator series follow the class definitions of the source analysis.

Table S8 Registered COVID-19 clinical trials cited for the compounds in Tables 2 and 3

NCT number	Compound	Study title	Conditions	Phase	Status	Study type	Enrolment	Interventions as registered	Single agent or combination	Sponsor
NCT04387240	Artesunate	Evaluating the Efficacy of Artesunate in Adults With Mild Symptoms of COVID-19	COVID-19	Phase 2	Withdrawn	Interventional	0	Artemisinin / artesunate; placebo	Single agent	Princess Nourah Bint Abdulrahman University
NCT05273242	Artesunate	Add-on Study on WHO Solidarity Trial Plus for COVID-19 in Nepal	COVID-19	Phase 4	Unknown	Interventional	400	Artesunate injection	Single agent	Nepal Health Research Council
NCT04330690	Artesunate	Treatments for COVID-19: Canadian Arm of the SOLIDARITY Trial	COVID-19	Phase 3	Unknown	Interventional	2900	Artesunate; imatinib; infliximab; dexamethasone; LSALT peptide	Single agent, one arm of a multi-arm study	Sunnybrook Health Sciences Centre
NCT04374019	Artesunate	Novel Agents for Treatment of High-risk COVID-19 Positive Patients	COVID-19	Phase 2	Terminated	Interventional	13	Ivermectin; camostat mesilate; Artemisia annua; artesunate	Single agent, one arm of a multi-arm study	Susanne Arnold
NCT04475107	Artesunate	The Efficacy and Safety of Pyramax in Mild to Moderate COVID-19 Patients	COVID-19	Phase 2	Completed	Interventional	113	Pyronaridine-artesunate; placebo	Fixed-dose combination	Shin Poong Pharmaceutical Co. Ltd.
NCT05084911	Artesunate	The Efficacy and Safety of Pyramax in Mild to Moderate COVID-19 Patients (Phase 3)	COVID-19	Phase 3	Completed	Interventional	1807	Pyronaridine-artesunate (Pyramax); placebo	Fixed-dose combination	Shin Poong Pharmaceutical Co. Ltd.
NCT05633420	Artesunate	Pilot Clinical Trial to Explore Efficacy and Safety of Pyramax in Mild to Moderate COVID-19 Patients	COVID-19	Phase 2	Completed	Interventional	20	Pyronaridine-artesunate (Pyramax)	Fixed-dose combination	Shin Poong Pharmaceutical Co. Ltd.
NCT04701606	Artesunate	The Safety and Efficacy of Pyronaridine-artesunate in COVID-19 Patients	COVID-19	Phase 2/3	Unknown	Interventional	402	Pyronaridine-artesunate (Artecom); placebo	Fixed-dose combination	Shin Poong Pharmaceutical Co. Ltd.
NCT04532931	Artesunate	COVID-19 Treatment in South Africa	COVID-19	Phase 2	Completed	Interventional	192	Standard of care; artesunate-amodiaquine; pyronaridine-artesunate; favipiravir plus nitazoxanide; sofosbuvir/daclatasvir	Fixed-dose combination, one arm of a multi-arm study	Shin Poong Pharmaceutical Co. Ltd.
NCT04695197	Artesunate	Malaria as a Risk Factor for COVID-19 in Western Kenya and Burkina Faso	COVID-19; malaria	Phase 3	Completed	Interventional	143	Artemether-lumefantrine; pyronaridine-artesunate	Fixed-dose combination	Liverpool School of Tropical Medicine
NCT04461340	Sirolimus	Efficacy and Safety of Sirolimus in COVID-19 Infection	COVID-19	Phase 2	Unknown	Interventional	40	Sirolimus	Single agent	Alexandria University
NCT04341675	Sirolimus	Sirolimus Treatment in Hospitalized Patients With COVID-19 Pneumonia	COVID-19	Phase 2	Completed	Interventional	32	Sirolimus; placebo	Single agent	University of Cincinnati
NCT04371640	Sirolimus	Sirolimus in COVID-19 Phase 1	COVID-19	Phase 1	Withdrawn	Interventional	0	Sirolimus; placebo	Single agent	Walter K. Kraft
NCT04482712	Sirolimus	Effects of mTOR Inhibition With Sirolimus in Patients With COVID-19 to Moderate the Progression of ARDS	ARDS; respiratory failure; SARS-CoV-2	Phase 1/2	Withdrawn	Interventional	0	Rapamycin; placebo	Single agent	The University of Texas Health Science Center at San Antonio
NCT04374903	Sirolimus	Hydroxychloroquine in Combination With Sirolimus and Dexamethasone for Treating COVID-19 Patients	COVID-19	Not applicable	Withdrawn	Interventional	0	Hydroxychloroquine and azithromycin versus hydroxychloroquine and sirolimus	Combination arm	King Hussein Cancer Center
NCT04948203	Sirolimus	Assessing the Efficacy of Sirolimus in Patients With COVID-19 Pneumonia for Prevention of Post-COVID Fibrosis	Pulmonary fibrosis; COVID-19 pneumonia; long COVID	Phase 2/3	Recruiting	Interventional	60	Sirolimus	Single agent	University of Chicago
NCT06257420	Sirolimus	Low Dose Rapamycin in ME/CFS, Long-COVID, and Other Infection Associated Chronic Conditions	Myalgic encephalomyelitis; long COVID	Not applicable	Enrolling by invitation	Observational	150	Rapamycin	Single agent	Simmaron Research Inc.

Note. Records were retrieved from ClinicalTrials.gov and checked against the registry entry; only records that list the compound among the registered interventions are included.

Table S9 Significantly down-regulated gene sets in cilastatin-treated HT29 cells (LINCS 2020)

Gene set	NES	FDR
GOMF SNORNA BINDING	-3.76	0.043
GOBP RIBONUCLEOPROTEIN COMPLEX BIOGENESIS	-3.45	0.043
GOBP RIBOSOME BIOGENESIS	-3.43	0.043
GOBP RRNA METABOLIC PROCESS	-3.36	0.043
GOBP NCRNA PROCESSING	-3.36	0.043
GOMF U3 SNORNA BINDING	-3.33	0.043
GOBP SPLICEOSOMAL COMPLEX ASSEMBLY	-3.21	0.043
GOBP NCRNA METABOLIC PROCESS	-3.09	0.043
GOCC SPLICEOSOMAL COMPLEX	-3.09	0.043
GOBP POSITIVE REGULATION OF MRNA PROCESSING	-3.07	0.043
GOMF TRANSLATION TERMINATION FACTOR ACTIVITY	-3.00	0.043
GOCC INTRINSIC COMPONENT OF MITOCHONDRIAL INNER MEMBRANE	-2.99	0.043
GOBP RIBONUCLEOPROTEIN COMPLEX SUBUNIT ORGANIZATION	-2.99	0.043
GOBP RNA SPLICING VIA TRANSESTERIFICATION REACTIONS	-2.97	0.043
GOBP RNA PROCESSING	-2.97	0.043
GOCC U2 TYPE SPLICEOSOMAL COMPLEX	-2.91	0.043
GOBP RNA POLYADENYLATION	-2.86	0.043
GOBP POSITIVE REGULATION OF CHROMOSOME ORGANIZATION	-2.79	0.043
GOMF RNA BINDING	-2.77	0.043
GOBP PROTEIN LOCALIZATION TO MITOCHONDRION	-2.74	0.043
GOBP POSITIVE REGULATION OF TELOMERE MAINTENANCE	-2.74	0.043
GOBP PROTEIN TARGETING TO MITOCHONDRION	-2.73	0.043
GOCC INTRINSIC COMPONENT OF MITOCHONDRIAL MEMBRANE	-2.73	0.043
GOMF LIGASE ACTIVITY FORMING CARBON SULFUR BONDS	-2.66	0.043
GOBP MRNA PROCESSING	-2.64	0.043
GOMF ENDODEOXYRIBONUCLEASE ACTIVITY	-2.58	0.043
GOBP REGULATION OF CELLULAR MACROMOLECULE BIOSYNTHETIC PROCESS	-2.26	0.043
GOCC MITOCHONDRION	-2.22	0.043
GOBP REGULATION OF DNA METABOLIC PROCESS	-1.94	0.043
GOBP ORGANONITROGEN COMPOUND CATABOLIC PROCESS	-1.71	0.043
GOBP MRNA SPLICE SITE SELECTION	-3.23	0.11
GOMF PHOSPHATIDIC ACID TRANSFER ACTIVITY	-3.07	0.11
GOCC PRERIBOSOME	-3.44	0.13
GOCC RIBONUCLEOPROTEIN COMPLEX	-2.78	0.13
GOBP RNA DEPENDENT DNA BIOSYNTHETIC PROCESS	-2.59	0.13
GOCC SPLICEOSOMAL SNRNP COMPLEX	-3.06	0.15
GOMF UBIQUITIN LIKE MODIFIER ACTIVATING ENZYME ACTIVITY	-3.12	0.18
GOBP NCRNA 3 END PROCESSING	-3.03	0.18
GOMF METAL CLUSTER BINDING	-2.86	0.18
GOCC MITOCHONDRIAL MATRIX	-2.73	0.18
HP SPASTIC DIPLEGIA	-2.49	0.18
GOBP REGULATION OF DNA BIOSYNTHETIC PROCESS	-2.31	0.18

Note. NES indicates the normalized enrichment score, with negative values reflecting down-regulation.
Of the 42 gene sets, 30 also satisfy a false discovery rate below 0.05.

Table S10 Disease queries that rank the 17 drugs used or evaluated in COVID-19 higher than the COVID-19 query does

Rank of the query	Disease	Median rank of the 17 drugs (drug nodes)	Interquartile range	Number of the 17 with an edge to the disease
1	Cryopyrin-Associated Periodic Syndromes	57	24-328	1
2	Rheumatoid Arthritis	65	26-209	8
3	Synovitis	65	36-233	3
4	Polymyalgia Rheumatica	69	56-354	1
5	Ankylosing spondylitis	75	35-412	4
6	Arthritis, Psoriatic	78	37-410	4
7	Multiple Sclerosis, Relapsing-Remitting	79	47-489	1
8	TNF receptor-associated periodic fever syndrome (TRAPS)	82	31-376	0
9	Tenosynovitis	88	39-380	4
10	Lupus Erythematosus, Systemic	88	17-286	5
11	Scleroderma	102	29-458	0
12	Musculoskeletal Diseases	102	58-370	4
13	Pelvic Inflammatory Disease	105	37-399	0
14	Myasthenia Gravis	106	67-592	0
15	Inflammatory Bowel Diseases	109	51-660	4
16	Degenerative polyarthritis	115	59-571	0
17	Thyroiditis	116	35-506	4
18	Arthritis, Gouty	124	27-421	4
19	Crohn Disease	126	19-686	5
20	Urticaria	131	81-626	0
21	Ulcerative Colitis	132	98-841	2
22	COVID-19	135	44-379	0

Note. The 17 drugs are those used or evaluated in COVID-19 that are present in the graph; they are listed with their ranks for the COVID-19 query in Table S3.

All 240 disease queries of the retrieval evaluation, the 239 RepoDB diseases and COVID-19, were ordered by the median rank of these 17 drugs among the 4314 drug nodes. The 21 queries placed above COVID-19 are listed here, with COVID-19 itself in the last row for comparison; it is 22nd of 240.

The last column gives how many of the 17 drugs carry a drug–disease edge to that disease in the graph.

Table S11 ATC codes used to identify the candidate drugs in the reporting database

Drug	ATC code	ATC term
Cilastatin	J01DH51	Imipenem and cilastatin
	J01DH56	Imipenem, cilastatin and relebactam
Megestrol	G03AA04	Megestrol and ethinylestradiol
	G03AB01	Megestrol and ethinylestradiol
	G03AC05	Megestrol
	G03DB02	Megestrol
	G03FA08	Megestrol and estrogen
	G03FB04	Megestrol and estrogen
	L02AB01	Megestrol
Drotrecogin alfa (activated)	B01AD10	Drotrecogin alfa (activated)
Ethacrynic acid	C03CC01	Etacrynic acid

Note. Drugs were identified by ATC code rather than by free-text drug name. A report was counted as exposed if any of the listed codes appeared in it.

Cilastatin has no single-agent product; both codes are fixed-dose combinations with imipenem.

The ATC term for C03CC01 is spelled "etacrynic acid" in the classification; the manuscript uses the INN spelling "ethacrynic acid".

Table S12 Preferred terms grouped under the MedDRA High Level Term "Coronavirus infections"

Level	Code	Term
SOC	10021881	Infections and infestations
HLGT	10047438	Viral infectious disorders
**HLT**	**10084510**	**Coronavirus infections**
PT	10051905	Coronavirus infection
PT	10061982	Severe acute respiratory syndrome
PT	10075271	Middle East respiratory syndrome
PT	10084268	COVID-19
PT	10084380	COVID-19 pneumonia
PT	10084381	Coronavirus pneumonia
PT	10084451	Suspected COVID-19
PT	10084639	SARS-CoV-2 sepsis
PT	10084640	SARS-CoV-2 viraemia
PT	10085076	HCoV-229E infection
PT	10085077	HCoV-OC43 infection
PT	10085078	HCoV-NL63 infection
PT	10085079	HCoV-HKU1 infection
PT	10085080	Congenital COVID-19
PT	10085492	Vaccine derived SARS-CoV-2 infection
PT	10085503	Post-acute COVID-19 syndrome
PT	10086861	Breakthrough COVID-19

Note. MedDRA/J Ver. 27.1J. The 17 preferred terms under the High Level Term 10084510 define the outcome group used in the disproportionality analysis.
