# Supplementary material for: Multilayered Prioritization of Graph Attention Autoencoder–Derived Drug Repurposing Candidates for COVID-19
Source: Pharmaceuticals (Basel). 2026 Sep 25;19(10):1522. doi: 10.3390/ph19101522 (PMC13708601; doi:10.3390/ph19101522)
Supplement: Supplementary file 1 [file pharmaceuticals-19-01522-s001.zip › Supplementary Figures.pdf]

[Supplemental Figures]

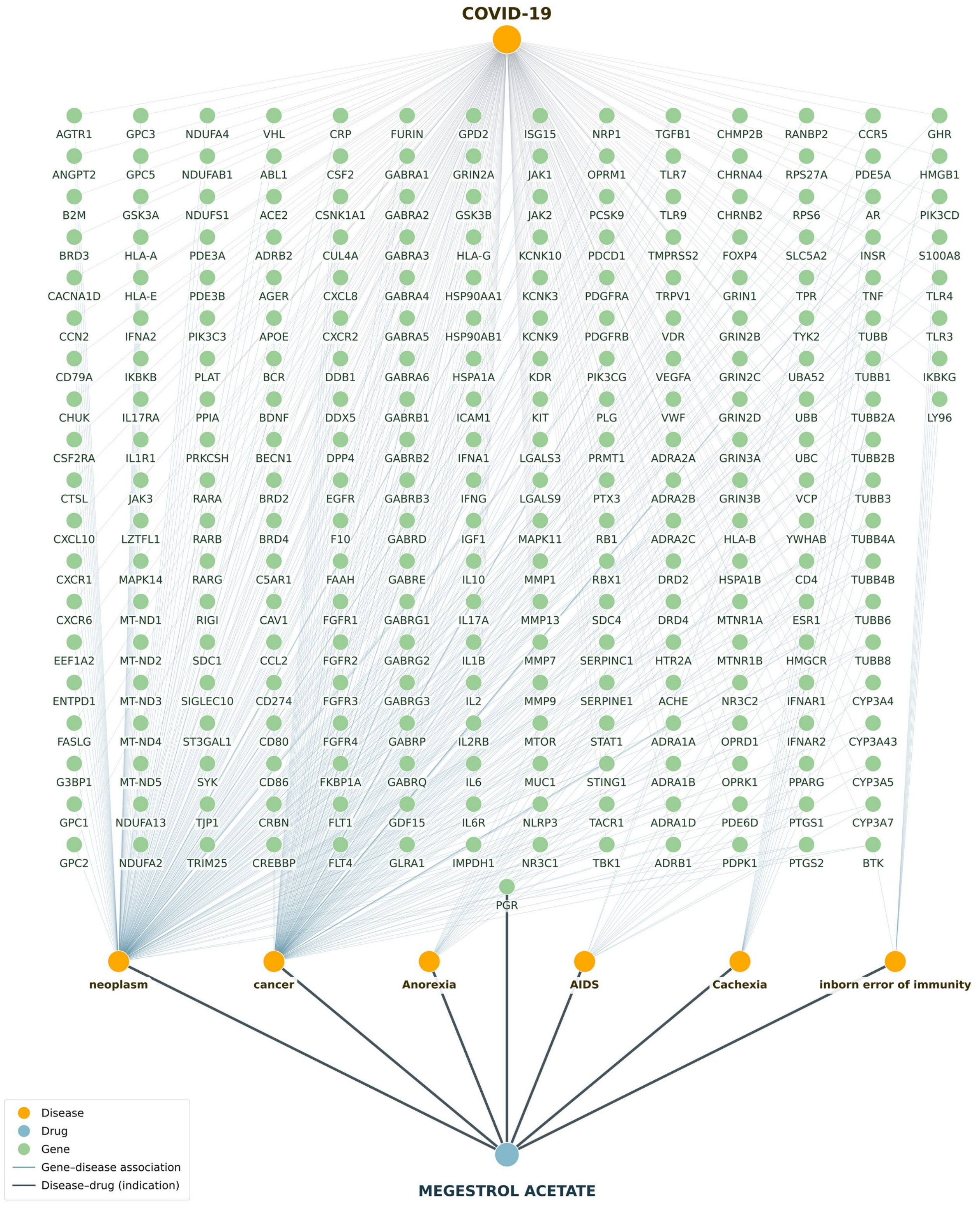

Figure S1 Subnetwork connecting COVID-19 and megestrol in multimodal knowledge graph

The subnetwork comprises the nodes on the shortest paths between the COVID-19 and megestrol (megestrol acetate) nodes; the two share no direct edge and are linked indirectly through 255 genes shared with six megestrol-indicated diseases (neoplasm, cancer, AIDS, anorexia, cachexia, and inborn error of immunity). The megestrol target PGR also lies on a shortest path (COVID-19–ESR1–PGR–megestrol). Nodes are colored by type: orange for diseases, blue for drugs, and green for genes.

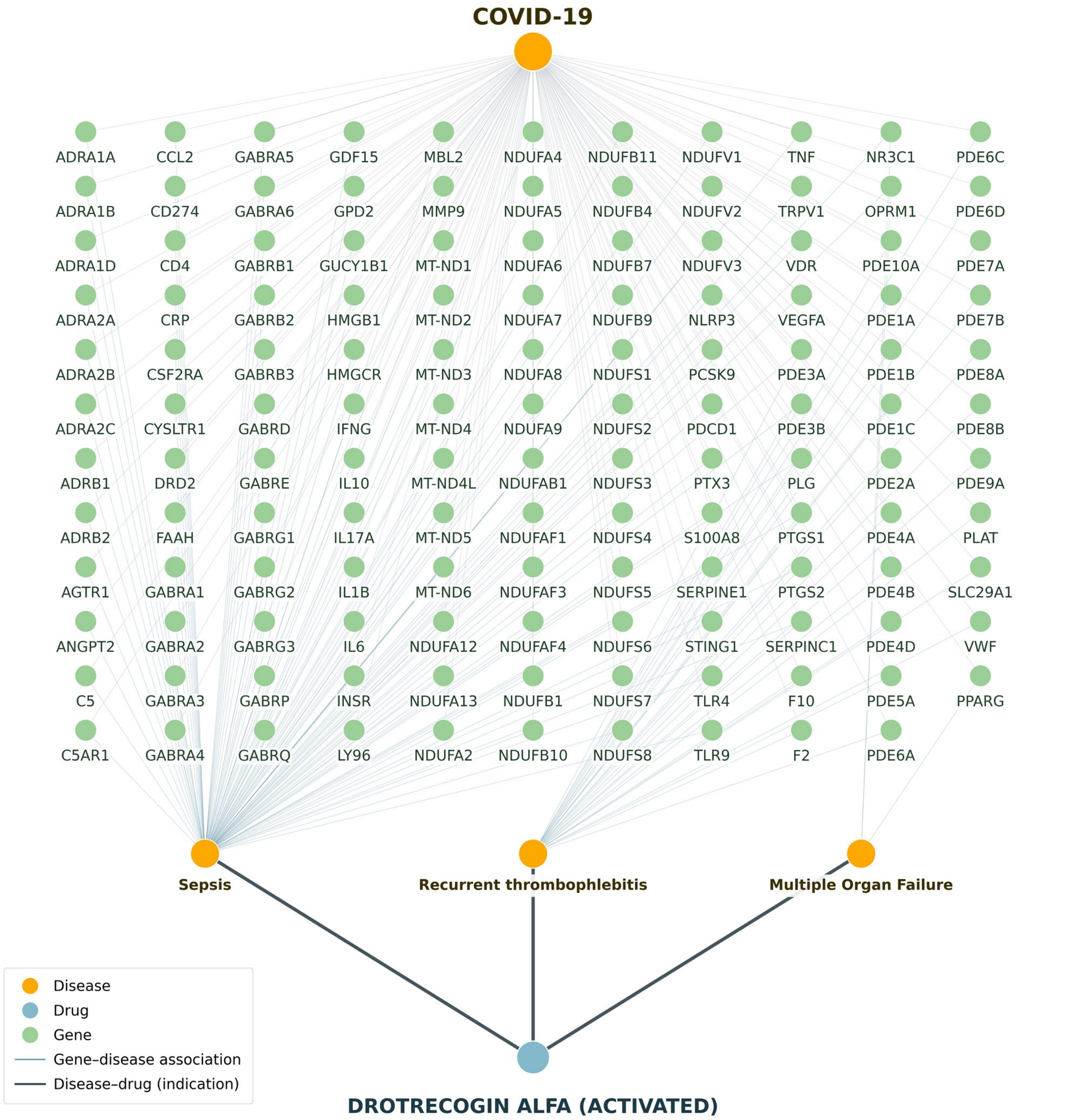

Figure S2 Subnetwork connecting COVID-19 and drotrecogin alfa in multimodal knowledge graph

The subnetwork comprises the nodes on the shortest paths between the COVID-19 and drotrecogin alfa (activated) nodes; the two share no direct edge and are linked indirectly through 131 genes shared with three drotrecogin alfa-indicated diseases (sepsis, multiple organ failure, and recurrent thrombophlebitis). Nodes are colored by type: orange for diseases, blue for drugs, and green for genes.

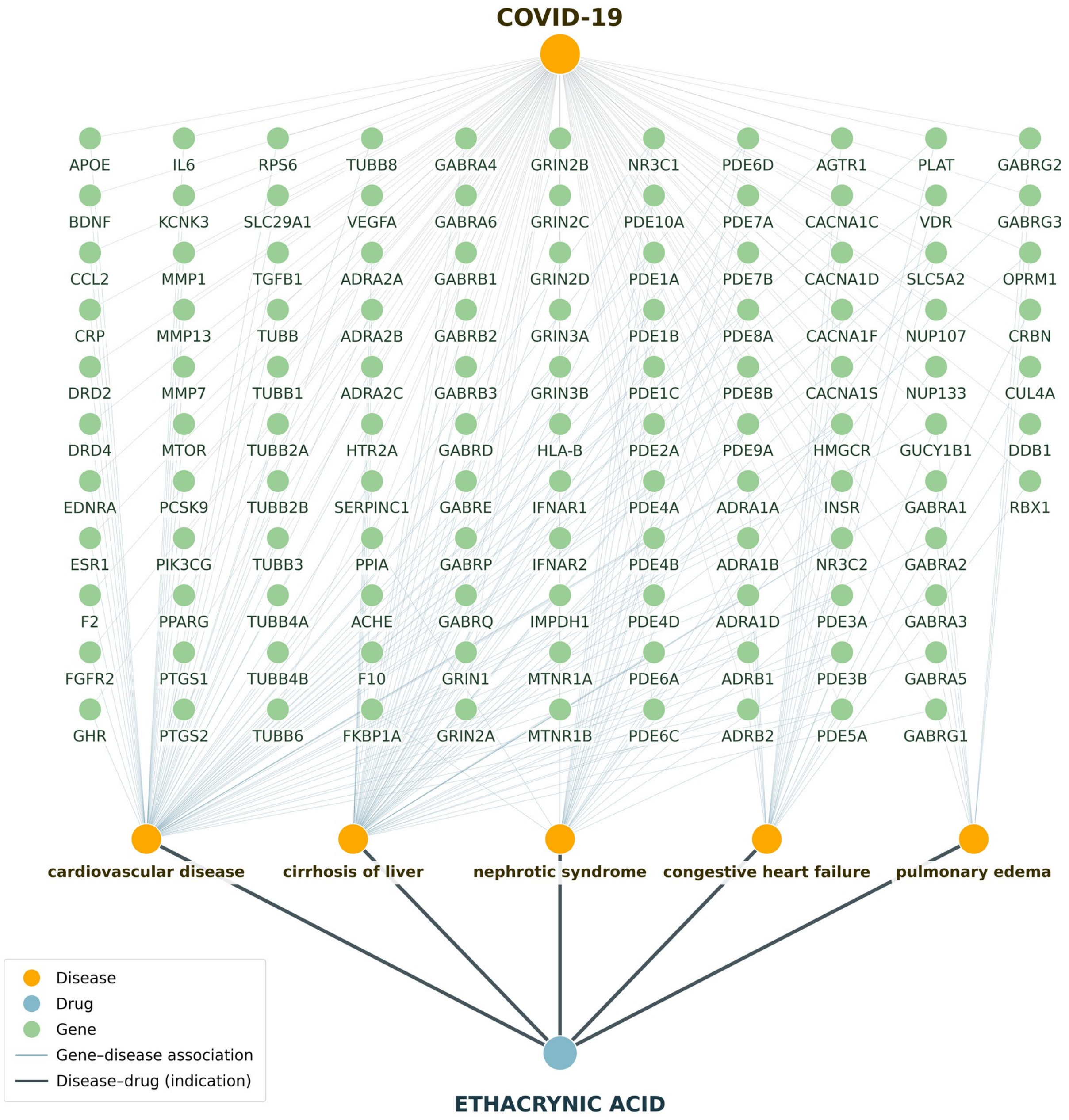

Figure S3 Subnetwork connecting COVID-19 and ethacrynic acid in multimodal knowledge graph

The subnetwork comprises the nodes on the shortest paths between the COVID-19 and ethacrynic acid nodes; the two share no direct edge and are linked indirectly through 117 genes shared with five ethacrynic acid-indicated diseases (cardiovascular disease, cirrhosis of liver, nephrotic syndrome, congestive heart failure, and pulmonary edema). Nodes are colored by type: orange for diseases, blue for drugs, and green for genes.

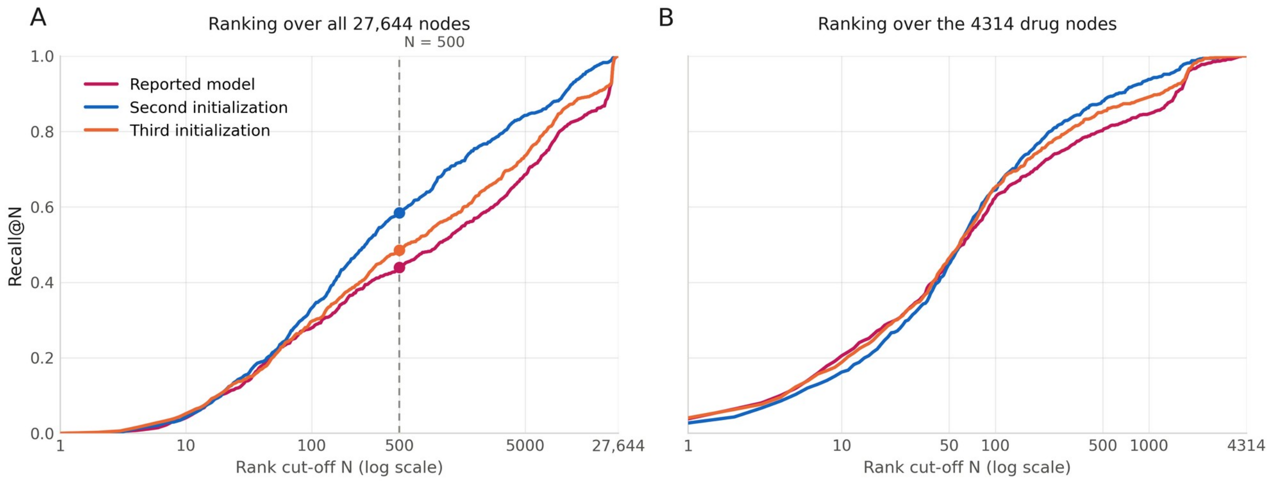

Figure S4 Retrieval of unlinked approved indications by three models trained from independent initializations

Recall@N is plotted against the rank cutoff N, for the 807 approved drug–disease indications in repoDB that span 239 diseases and that carry no corresponding edge in the graph. (A) Ranking over all 27,644 nodes of the graph; the dashed line marks the top-500 cutoff used in this study. (B) Ranking over the 4314 drug nodes alone. The model reported in this study and two models trained from independent initializations are shown, and the same candidate-identification procedure was applied to each.

A

Drug	Cases	Non-cases	ROR	95% CI
cilastatin	6	1716	0.19	0.09–0.43
imipenem	14	2422	0.32	0.19–0.53
meropenem	235	8096	1.59	1.39–1.81
ertapenem	11	1578	0.38	0.21–0.69
doripenem	4	126	1.73	0.64–4.69
biapenem	2	59	1.85	0.45–7.57

B

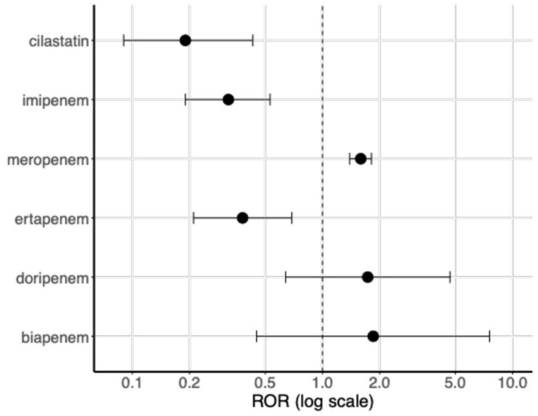

C

Drug	Cases	Non-cases	ROR	95% CI
cilastatin	6	1716	0.19	0.09–0.43
clavulanic acid	71	4462	0.87	0.69–1.10
sulbactam	34	1720	1.08	0.77–1.51
tazobactam	287	12,031	1.30	1.16–1.46
avibactam	15	584	1.40	0.84–2.34
sultamicillin	3	186	0.88	0.28–2.75

D

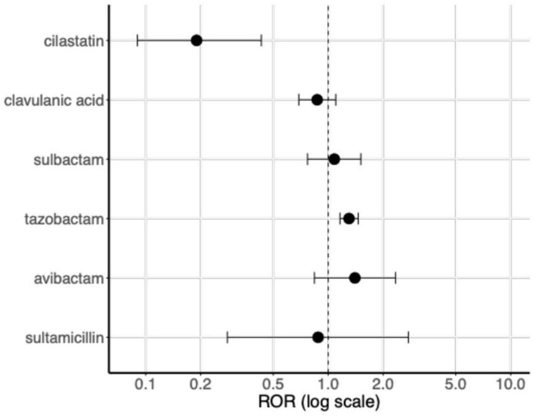

Figure S5 DPA of carbapenems and of β-lactamase inhibitors, and coronavirus infections

(A) RORs and 95% CIs for cilastatin and other carbapenems. (B) Forest plot visualizing RORs for the same set of drugs. (C) RORs and 95% CIs for cilastatin and β-lactamase inhibitors. (D) Forest plot visualizing RORs for the same set of drugs. Complete 2 × 2 counts for these drugs are given in Table S7.

A

Drug	Cases	Non-cases	ROR	95% CI
megestrol	24	2077	0.63	0.42–0.94
sex hormone	332	34,697	0.52	0.47–0.58
anticancer agent	52	4594	0.62	0.47–0.81
systemic hormonal contraceptive	283	31,764	0.48	0.43–0.54
progesterone	117	19,510	0.33	0.27–0.39

B

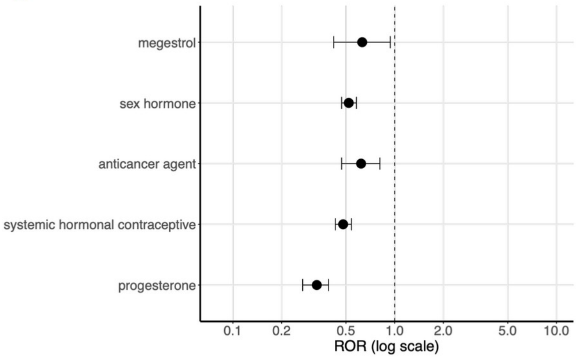

Figure S6 DPA of progestins and related drug classes and coronavirus infections

(A) RORs and 95% CIs for progestins and related drug classes. (B) Forest plot visualizing RORs for the same set of drugs. Complete 2 × 2 counts for these classes are given in Table S7.
